# Integrative cross‐tissue analysis unveils complement‐immunoglobulin augmentation and dysbiosis‐related fatty acid metabolic remodeling during mammalian aging

**DOI:** 10.1002/imt2.70027

**Published:** 2025-04-12

**Authors:** Feng Zhang, Rong Li, Yasong Liu, Jinliang Liang, Yihang Gong, Cuicui Xiao, Jianye Cai, Tingting Wang, Qiang You, Jiebin Zhang, Haitian Chen, Jiaqi Xiao, Yingcai Zhang, Yang Yang, Hua Li, Jia Yao, Qi Zhang, Jun Zheng

**Affiliations:** ^1^ Department of Hepatic Surgery and Liver Transplantation Center of the Third Affiliated Hospital of Sun Yat‐sen University; Organ Transplantation Research Center of Guangdong Province Guangdong Province Engineering Laboratory for Transplantation Medicine Guangzhou China; ^2^ Guangdong Provincial Key Laboratory of Liver Disease Research the Third Affiliated Hospital of Sun Yat‐sen University Guangzhou China; ^3^ Biotherapy Center & Cell‐gene Therapy Translational Medicine Research Center the Third Affiliated Hospital of Sun Yat‐sen University Guangzhou China; ^4^ Department of Anesthesiology the Third Affiliated Hospital of Sun Yat‐sen University Guangzhou China; ^5^ Comprehensive Transplant Center, Feinberg School of Medicine Northwestern University Chicago USA; ^6^ Department of Hepatobiliary Surgery People's Hospital of Xinjiang Uyghur Autonomous Region Urumqi China

**Keywords:** aging, complement system, gut microbiota dysbiosis, immunoglobulin, lipid metabolic remodeling, polyunsaturated fatty acids

## Abstract

Aging‐related decline and adaptation are complex, multifaceted processes that affect various tissues and increase risk of chronic diseases. To characterize key changes in cross‐tissue aging, we performed comprehensive proteomic and metabolomic analyses across 21 solid tissues and plasma samples, alongside shotgun metagenomic profiling of fecal microbial communities in young and aged mice. Our findings revealed widespread aging‐rewired chronic inflammation, characterized by complement system activation in plasma and universal immunoglobulins accumulation across multiple solid tissues. This inflammatory remodeling significantly enhanced vulnerability to aging‐related tissue injury. Moreover, we identified organ‐specific and organ‐enriched proteins with high functional specificity. Among these, aging‐related proteins were closely linked to disorders arising from lipid metabolism dysfunction. Analysis of multi‐tissue metabolomic and fecal metagenomic profiles revealed that aging significantly disrupted inter‐tissue metabolic coupling, activities of polyunsaturated fatty acids metabolism, and gut microbiota homeostasis. Aged mice exhibited a marked decrease in *Escherichia* and an increase in *Helicobacter*, strongly correlating with alterations in omega‐3 and omega‐6 fatty acid abundances. Through multi‐omics integration, we identified key molecular hubs driving organismal responses to aging. Collectively, our study uncovers extensive aging‐associated alterations across tissues, emphasizing the interplay between systemic inflammation and dysbiosis‐driven fatty acid remodeling. These findings provide deeper insights into the development of healthy aging from a cross‐tissue perspective.

## Highlights

- Integrated proteomic, metabolomic, and metagenomic analyses revealed tissue‐spanning and tissue‐specific changes during mammalian aging.
- Systemic inflammaging was triggered by the accumulation of immunoglobulins in solid tissues and the activation of circulating complement system.
- Aging‐related remodeling of tissue‐resolved proteostasis and fatty acid pathways contributed to disorders related to lipid metabolism dysfunction.
- Dysbiosis of gut microbiota, involving *Escherichia* and *Helicobacter*, was significantly associated with dysregulated metabolism of polyunsaturated fatty acids.

## Graphical abstract

Our study provides a comprehensive multi‐omics profile of aging, integrating proteomic, metabolomic, and metagenomic analyses across diverse tissues and plasma. We identified the synergistic amplification of the circulating complement system and tissue‐wide immunoglobulin accumulation as key molecular drivers of inflammaging. Furthermore, we uncovered gut microbiota dysbiosis, characterized by a significant decrease in *Escherichia* and an increase in *Helicobacter*, which contributes to lipid metabolic reprogramming, particularly the dysregulation of polyunsaturated fatty acid metabolism across multiple tissues. By elucidating the intricate interplay between inflammaging and dysbiosis‐induced fatty acid metabolic remodeling in mammalian aging, our study highlights promising gero‐protective targets and pathways that could mitigate aging‐related diseases and enhance health span. These insights pave the way for developing novel therapeutic strategies to promote healthy aging.

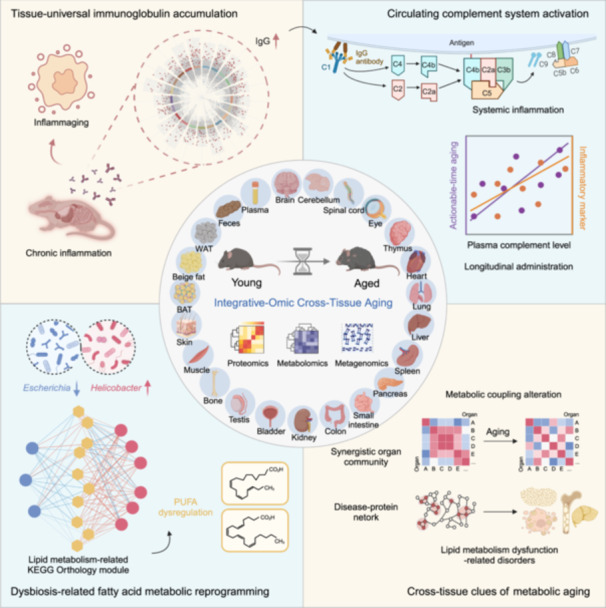

## INTRODUCTION

Living organisms display a sophisticated organization throughout their lifetimes, characterized by intricate system‐wide coordination and specialized organ‐specific compartmental functions [1, 2, 3]. Aging, a complex physiological process, strongly correlates with the risk of chronic illnesses such as metabolic disorders, cardiovascular diseases, and neurodegenerative conditions [4]. This process involves a multiorgan aging network, where primary organ aging progresses to affect multiple systems [5, 6]. Although several traits, like chronic inflammation, metabolic reprogramming, and dysbiosis, are known to contribute to age‐related decline [7, 8, 9], the relationships between them are unclear. A systematic investigation into multiorgan aging is necessary to obtain a panoramic view of aging‐derived disturbance in trajectories and identify potential gero‐protective targets for healthy aging and longevity.

Recent advances in high‐throughput omics technologies has enabled comprehensive characterization of molecular changes associated with aging, revealing tissue‐wide, cell‐specific, and spatially organized transcriptomic alterations, such as unfolded protein binding and inflammatory/immune responses [1, 10, 11]. Indeed, research on aging‐related proteomic changes can elucidate the loss of proteostasis and discrepancies between protein and transcript changes, including decreased proteasome activity and ribosome occupancy [12, 13]. Accordingly, the plasma proteome offers a comprehensive circulating reflection of various aspects across different cell types and tissues [10, 14]. A longitudinal human cohort study, based on plasma omics profiling, identified consistent nonlinear patterns in aging‐related molecular markers, highlighting significant dysregulation in carbohydrate, lipid, and alcohol metabolism [15]. Despite these advances, the precise correlations between plasma and tissue proteins, as well as their metabolic interplay, remain poorly understood. Thus, deeper explorations are needed to disentangle the link between circulating and parenchymal proteomics and the interactive molecular hubs between proteomics and metabolomics from a cross‐tissue perspective.

Chronic inflammation and metabolic reprogramming have been reported as the predominantly significant processes leading to aging‐related nonlinear dynamics [15]. Elderly individuals susceptible to inflammaging often exhibit vulnerability to multiple metabolic disorders, like type 2 diabetes [16]. Identifying critical tissue‐specific or ‐universal molecules related to inflammaging is crucial for alleviating aging‐related metabolic diseases. However, these processes are greatly influenced by the gut microbe ecosystem, where diverse microbial species regulate host metabolism primarily through their components and metabolic byproducts. For instance, *Bacteroides fragilis* is capable of producing short‐chain fatty acids (SCFAs) and indole derivatives, which promote anti‐inflammatory responses by modulating the differentiation of Th17 and Treg cells [17]. However, the interpretation of integrative species‐metabolites in multiorgan aging remains unexplored. Further studies are encouraged to eliminate the chasm in our understanding of the causal relationship and cross‐tissue metabolic coupling between gut microbes and aging.

In this study, we delineated the integrative molecular hubs in aged mice by profiling the comprehensive proteome and metabolome across a wide range of tissues, along with shotgun metagenomic analysis on microbial communities from fecal samples. Through in‐silico analysis and ex‐vivo validation, we characterized both tissue‐ubiquitous and tissue‐specific alterations during aging and identified the co‐augmentation of circulating complement system and parenchymal immunoglobulin as key molecular nodes of inflammaging. Besides, we revealed dysbiosis‐triggered lipid metabolism dysfunction (LMD), especially the remodeling of fatty acid metabolism derived from a decrease in *Escherichia* and an increase in *Helicobacter*. In total, these findings provided new insights for monitoring lipid metabolism disturbance‐related diseases and for intervening in the cross‐tissue aging process to achieve healthy aging.

## RESULTS

### Proteomic and metabolomic characterization of mammalian aging across multiple tissues

**Figure 1:**
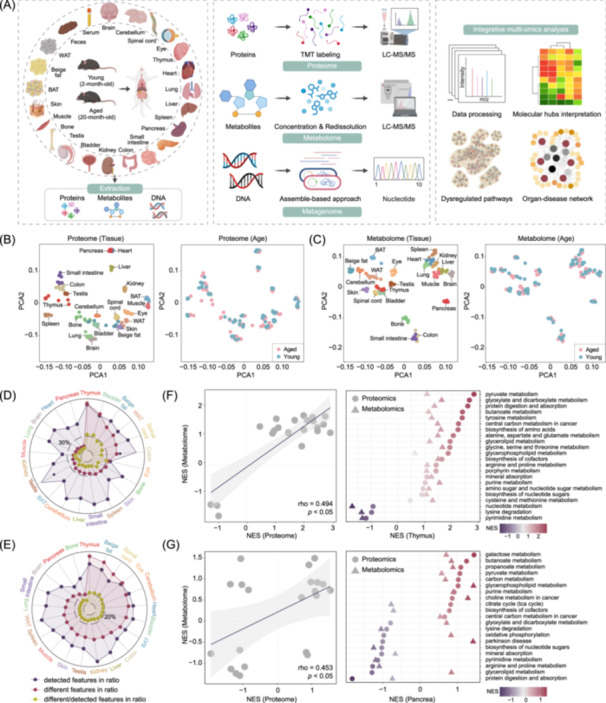
Characterization of age‐related cross‐tissue proteome and metabolome in mammals. (A) Schematic illustration of experimental design for tissue sampling, omics sequencing and integrative analysis in the young and aged mice. BAT, brown adipose tissues; LC‐MS, liquid chromatography‐mass spectrometry; TMT, tandem mass tags; WAT, white adipose tissues. (B) Principal component analyses (PCA) plot showing proteomic clustering based on tissue (left) or age (right). *n* = 4 mice per group. (C) PCA plot showing metabolomic clustering based on tissue (left) or age (right). *n* = 4 mice per group. Relative ratios of the number of detected, differential and detected/differential proteins (D) or metabolites (E) across tissues. Left: Scatterplots of the normalized enrichment scores (NES) for features from proteomic and metabolomic pathway enrichment analyses in the thymus (F) or pancreas (G). Spearman correlations were calculated. The area shaded around the regression line indicates the 95% confidence interval. Right: Significant pathway terms enriched in both proteome (circles) and metabolome (triangles) for young (dark purple) or aged (deep red) thymus (F) or pancreas (G).

To investigate the proteomic and metabolomic alterations in aging across various organs, we explored 21 tissues from C57BL/6J male mice at both young (2 months) and aged (20 months) stages (*n* = 4 mice per group), covering 10 key biological systems. The selected systems contained a spectrum of essential organs, including heart, lung, liver, colon, small intestine, pancreas, spleen, kidney, bladder, testis, whole brain, thymus, spinal cord, cerebellum, bone, muscle, skin, brown adipose tissue (BAT), white adipose tissue (WAT), beige fat, and eye. Furthermore, plasma samples were acquired for proteomic profiles, thereby providing a panoramic view of circulating‐local aging‐related effects (Figure 1A).

Proteomic profiling was performed using isobaric tandem mass tags (TMT). The medians of coefficient of variation (CV) in biological replicate samples were less than 0.3 and remained relatively consistent across different organs in young and aged mice, indicating high data robustness [18] (Figure S1). In total, we identified 13,328 proteins, each supported by at least two unique peptides (FDR ≤ 1%, length ≥ 7 amino acids, mass accuracy ≤ 20 ppm, Andromeda score ≥ 40) (Figure S2A). Testis and thymus had the highest coverages, each containing over 8 × 10^3^ proteins and 8 × 10^4^ peptides. In contrast, skin, muscle, and eye showed substantially lower counts, implying a tissue‐specific bias in proteomic profiling (Figure S2B). Furthermore, we mapped the subcellular localization of proteins and discovered that most identified proteins were from the nucleus (*n* = 4007) and cytoplasmic matrix (*n* = 3032), followed by plasma membrane (*n* = 1983) and extracellular matrix (*n* = 1932) (Figure S2C).

For metabolomic profiling, we employed liquid chromatography‐mass spectrometry (LC‐MS) in conjunction with hydrophilic interaction chromatography (HILIC) to separate polar and ionizable metabolites. Across the datasets, we identified 9399 distinct annotated metabolites and defined their chemical superclass based on the ClassyFire classification system [19] (Figure S2D). Lipids and lipid‐like molecules constituted the largest proportion at 40.32%, primarily comprising complex lipids such as glycerophospholipids (GP), saccharolipids (SL), phospholipids (PL), sterol lipids (Ste), and glycerolipids (GL). Fatty acids, categorized as simple lipids, also showed significant changes during aging. Figure S2E provides tissue‐resolved metabolite counts by classes. To assess potential batch effects or run‐to‐run variations, we conducted principal component analyses (PCA) on the integrated proteomics and metabolomics data set. As expected, samples clustered predominantly by tissue rather than age, indicating that tissue‐specific characteristics outweighed age‐related differences in our study (Figure 1B,C).

Based on the ratio of age‐related to total detected proteins or metabolites in each tissue, we identified the thymus as a highly age‐divergent organ and the pancreas as an age‐conserved organ (Figure 1D,E). In the thymus, aging led to differential expression of 2790 proteins and 359 metabolites (Figure S2F), while the pancreas showed only 278 proteins and 93 metabolites (Figure S2G). Building upon these results, we performed gene set enrichment analysis of the Kyoto Encyclopedia of Genes and Genomes (KEGG) and found high pathway‐level consistency between proteomic and metabolomic profiles in both the thymus (Spearman, rho = 0.494, *p* < 0.05, Figure 1F) and the pancreas (Spearman, rho = 0.453, *p* < 0.05, Figure 1G). Specifically, omics‐consistent shifts were primarily observed in metabolic pathways, including enhanced glycerophospholipid metabolism, purine metabolism, and pyruvate metabolism, as well as reduced lysine degradation and pyrimidine metabolism (Figure 1F,G). Notably, dysregulation of purine metabolism was featured by the upregulation of ENTPD1, ENTPD4, and AK2 in the thymus, and ENTPD2 and AK4 in the pancreas (Figure S2F,G). Purine metabolism preserves mitochondrial function by inhibiting p53 activation to counter pancreatic β‐cell senescence, and it is essential for T‐cell differentiation, consistent with the observed decline in thymic regeneration and the deterioration of immunity with aging [20, 21, 22]. In the aged pancreas, galactose metabolism was significantly enriched, reflecting its protective effects against acinar necrosis and inflammation [23], and highlighting its involvement in low‐level inflammation associated with pancreatic aging [24]. Interestingly, the distinct enrichment of protein digestion and absorption pathway in the aged thymus (COL15A1, COL4A2) versus the young pancreas (CELA2A, CELA3B) further underscored the complexity and inter‐tissue heterogeneity of aging‐related effects (Figure S2F,G).

Taken together, we established a comprehensive age‐resolved proteomic and metabolomic resource spanning multiple tissues, laying the foundation for further systematic investigations.

### Diverse aging‐dependent proteome alterations across tissues

**Figure 2:**
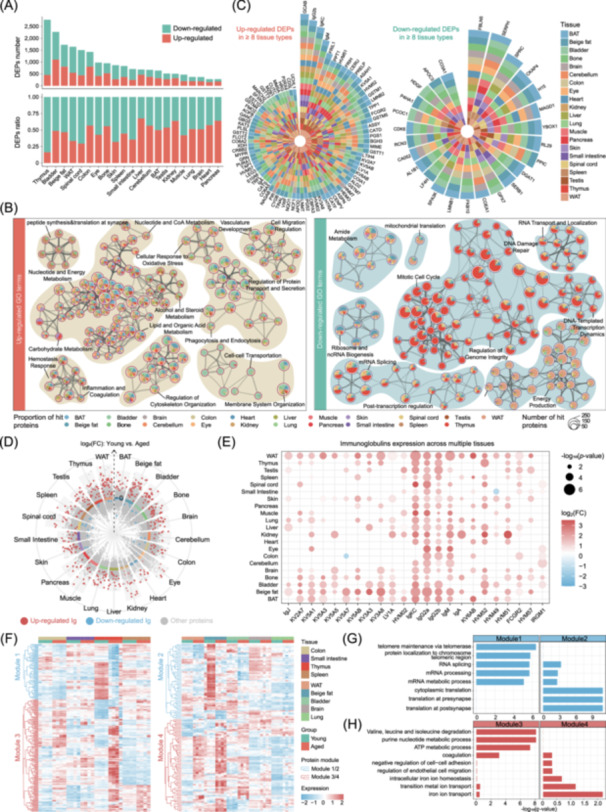
Characterized age‐dependent proteomic shifts across multiple tissues. (A) Bar plots showing the number (top) and ratio (bottom) of upregulated/downregulated differentially expressed proteins (DEPs) across 21 tissues. (B) Network plots indicating the enriched gene ontology (GO) terms/pathways of upregulated (left) and downregulated (right) DEPs. Each term is represented by a pie chart, where its size is proportional to the total number of proteins hits that fall into that specific term. The pie charts use color coding to represent different tissue identities, with the size of each slice indicating the percentage of proteins related to the respective term derived from the corresponding protein list. Terms with a similarity score > 0.3 are connected by edges, where edge thickness reflects the similarity score. (C) Radial plots showing high frequency upregulated (left) and downregulated (right) DEPs shared by at least eight tissue types. (D) Significance was determined by two‐sided Student's *t*‐tests for multiple proteins alteration between two groups, with upregulated (red) and downregulated (blue) immunoglobulin (Ig) labeled. (E) Bubble plot showing the expression of accumulated immunoglobulin across tissues during aging. (F) Heatmap of the four expression modules revealed by k‐means clustering in Cluster 1 (left) and Cluster 2 (right). The protein expression trends can be divided into two aging‐reduced modules, denoted Modules 1 and 2, and two aging‐accumulated modules, denoted Modules 3 and 4. Module‐specific and ‐shared GO terms/pathways between Module 1 and 2 (G), as well as Modules 3 and 4 (H).

To investigate tissue‐wide effects of differentially expressed proteins (DEPs), defined as proteins with significant expression changes between young and aged groups, we systematically cataloged the varying quantities and uneven‐distributed ratios of up‐/downregulated proteins across various tissues (Figure 2A). We observed considerable overlap among both aging‐associated DEPs across tissues, suggesting that certain aging‐related effects may be shared among different organs (Figure S3A).

Consistent with these observations, Gene Ontology (GO) analysis of aging‐accumulated proteins revealed tissue‐shared enrichment in lipid and organic acid metabolism, nucleotide metabolism, inflammation, and coagulation (Figure 2B, Figure S3B). Overlapping upregulated proteins further identified tissue interplay in monocarboxylic acid metabolism, lipid catabolism, and fatty acid catabolism (Figure S3B). In contrast, aging‐reduced proteins showed less inter‐tissue overlap, exemplified by genomic instability in the thymus (involving DNA damage repair, regulation of genome integrity and DNA‐templated transcription dynamics) and diminished energy production in the eye. However, amide metabolism remained a tissue‐shared pathway specifically enriched among aging‐reduced proteins (Figure 2B).

To further identify DEPs that were highly shared by multiple tissues, we identified high‐frequency DEPs with significant differences in at least eight tissues and ranked them by the number of tissues affected (Figure 2C). It was noteworthy that most high‐frequency upregulated DEPs were immunoglobulins (GCAB, IgG2b, IgKC, IgM, etc.), whereas no immunoglobulins or related proteins were detected among the high‐frequency downregulated DEPs (Figure 2C). Next, we explored immunoglobulin expression profiles across multiple organs and found them ubiquitously elevated in aged tissues (Figure 2D). Specifically, IgKC, IgG2A, IgG2B, and IgM were upregulated in almost all aged organs (Figure 2E). Notably, liver transcripts of immunoglobulin genes such as *Jchain, Ighm, Igha and Ighg2b* did not show significant age‐related changes, despite pronounced proteomic alterations [1]. This discrepancy underscores how proteomic analyses can reveal functional shifts not fully captured by transcriptomics. In line with our proteomic data, immunofluorescence (IF) staining confirmed an age‐dependent increase in IgG expression across various tissues in male mice (Figure S3C). Overall, these results point to a potential link between tissue‐wide inflammaging and the pervasive accumulation of immunoglobulins, suggesting that modulating immunoglobulin expression maybe crucial for mitigating age‐related decline.

Given that advanced biological aging often extends from an organ initially affected by disease to multiple systems, we next investigated potential “organ communities” exhibiting similar aging proteomic patterns, as this may guide an integrated approach to treat aging‐related diseases [5]. By correlating protein expression profiles, we identified two clusters of organs, each with Spearman's rank correlation coefficients greater than 0.60 (Figure S3D). The first cluster (Cluster 1), comprising the colon, small intestine, thymus, and spleen, contained 5621 co‐expressed proteins. The second cluster (Cluster 2), composed of the WAT, beige fat, bladder, brain, and lung, included 4077 co‐expressed proteins (Figure S3E).

Unsupervised k‐means clustering of these co‐expressed proteins in both young and aged groups revealed four modules (Figure 2F): two modules with proteins showing reduced expression in aging (Modules 1 and 2) and two modules with proteins showing elevated expression in aging (Modules 3 and 4). Notably, Modules 1 and 2 were enriched for impaired mRNA metabolism and processing, indicating the significance of restoring these processes for gero‐protection. Module 1 also showed specific enrichment in telomere maintenance and protein localization to telomeric region, highlighting the dominant role of genome instability in aging thymus and suggesting the importance of preserving genome homeostasis in Cluster 1. Meanwhile, Module 2 was featured by cytoplasmic translation, as well as translation at pre‐synapse and post‐synapse pathways, underscoring the regulatory role of neural transmission in the aging of Cluster 2 organs (Figure 2G). For Modules 3 and 4, the enrichment of amino acid and nucleotide metabolism (Module 3), iron ion transportation (Module 4), and shared endothelial function and cell interactions between both modules demonstrated a variety of dysfunctional processes and gero‐protective targets during aging, such as metabolic reprogramming, ferroptosis, endothelial protection (Figure 2H). Altogether, this comprehensive analysis illustrates the synchronized nature of aging across different organs and highlights key targets for potential interventions for mitigating age‐related decline.

### Inflammatory correlation between plasma proteins and organ‐derived proteins

Proteomic changes observed across multiple tissues highlighted the critical role of circulating plasma proteins as mediators of systemic aging effects. These proteins are well‐established indicators of biological age and health status, reflecting the interplay between systemic and tissue‐specific aging processes, and offer predictive potential for assessing the risk of aging‐related diseases [25, 26].

**Figure 3:**
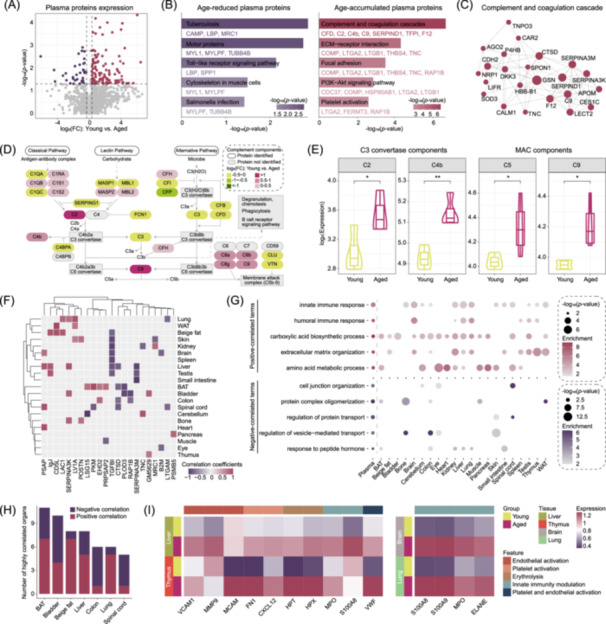
Significant correlation and tight inflammatory signaling linkage between plasma and organ proteins. Volcano plots (A) and GO enrichment analysis (B) for DEPs in the young versus aged plasma. (C) Protein‐protein interaction of aging‐related complement and coagulation cascade process. (D) Pathway visualization based on the Kyoto Encyclopedia of Genes and Genomes (KEGG) and complemented with details from the literature, with capsule blocks illustrating log_2_(FC) values of aged compared with young plasma. (E) Significant age differences of C3 convertase components (C2, C4b) and membrane attack complex (MAC) components (C5, C9) that varied in plasma. (F) Heatmap showing pivotal correlation (Spearman, |rho| > 0.6, *p* < 0.05) between plasma protein expression and corresponding organ‐derived protein expression. (G) GO enrichment of plasma‐tissue pairwise correlated proteins in plasma and various tissues. (H) The number of plasma‐tissue correlated proteins in the top seven organs. Positive Spearman correlations were labeled in the dark red and negative correlations in purple. (I) Aging‐related expression changes of markers of endothelial and platelet activation, erythrolysis and innate immunity modulation in liver, thymus, brain, and lung.

To explore the molecular connections between circulating proteins and aging in parenchymal tissues, we conducted a plasma proteome analysis. This analysis identified 1,243 plasma protein, of which 107 were upregulated, and 35 were downregulated in the aged mice compared to young controls (Figure 3A). Aging‐accumulated plasma proteins showed significant enrichment in pathways associated with complement and coagulation cascades, as well as PI3K‐AKT signaling pathway. Key proteins involved CFD, C2, C4b, C9, SERPIND1, CDC37, COMP, and ITGA2 (Figure 3B,C, Figure S4A).

The complement system, a cornerstone of the innate immune response, can be activated via the classical pathway (initiated by antigen‐antibody complexes), the lectin pathway (triggered by bacterial sugars), or the alternative pathway (spontaneously activated on cellular surfaces or pathogens). Our results revealed elevated components of the classical pathway (C1RA, C1QB, C1S1 and C1S2) and the C3 convertase proteins (C2 and C4b), accompanied by a reduction in the C1 inhibitor SERPING1. Additionally, the key complement component C5 was significantly upregulated in the aged group, associated with the recruitment of C6‐C9 (with C6 and C7 not detected) and the formation of the membrane attack complex (MAC), which mediates the activation or lysis of targeted cells (Figure 3D,E). Enzyme‐Linked Immunosorbent Assay (ELISA) confirmed elevated levels of C2, C4b, C5a, and soluble C5b‐9 (sC5b‐9) in the plasma of aged mice (Figure S4B). In contrast, complement proteins showed negligible accumulation across various tissues, indicating that the inflammatory response is predominantly activated within circulatory system rather than localized in specific organs (Figure S4C). Together, these results demonstrate a robust complement‐activated inflammatory response in aged plasma, primarily driven by the classical pathway via antigen‐antibody complexes.

To determine which organs and proteins contribute to age‐related proteomic linkages between plasma and organs, we calculated their trajectory correlations. We found that proteins related to immune activation and inflammatory response (IgJ, CD5L, LAC1, LV1A, SERPINA3K), extracellular matrix organization (POSTN, EHD2, GM5629, PSMB5) and nucleotide metabolism (PSAP) made significant contributions (Figure 3F, Figure S4D,E and Table S1). Enrichment analysis further confirmed that positive plasma‐tissue correlations in the brain, lung, liver and thymus were associated with innate and humoral immune responses, as well as extracellular matrix organization (Figure 3G). Additionally, BAT, bladder, beige fat, and liver showed the highest number of correlated tissues (Figure 3H).

Building upon these observations of systemic immunity and inflammatory response correlations between circulating plasma and parenchymal organs, we investigated the downstream effects of pathological complement activation in brain, lung, liver and thymus [27, 28, 29] (Figure 3I). The aged liver and thymus displayed elevated levels of endothelial and platelet activation, erythrolysis and innate immunity modulation, whereas the aged brain and lung were characterized by heightened neutrophil modulation (Figure 3I). Collectively, these plasma‐tissue interactions culminate in increased complement activity and inflammation‐driven tissue injury, offering new perspectives for the discovery of biomarkers and therapeutic interventions to mitigate aging‐related tissue injury.

To further investigate the systemic effects of complement proteins over time, we analyzed sequential plasma proteomic data from Stanford's cohort [4]. Individuals were sampled frequently over a defined actionable time frame (arbitrarily set at less than 2 years), allowing us to track personalized changes in healthy individuals. Among individuals with at least five healthy visits spanning 700 days to 2 years, most exhibited a positive correlation between the expression of various complement proteins and actionable‐time aging, independent of their baseline age (Figure S5A). Within this time frame, individuals with elevated levels of complement proteins often displayed increased clinical markers of inflammation. For instance, individual ZKVR426, who had nine visits within the 2‐year period, demonstrated a significant association between C1QA/C1QC and IgM/white blood cell (WBC) counts (Figure S5B,C). Similarly, individual ZK112BX, who had ten visits within the 2‐year period, displayed a significant association between C5/C9 and Neutrophils (NEUT)/WBC counts (Figure S5D,E). These findings highlight the significance of complement protein expression as a potential biomarker for monitoring age‐related changes and inflammatory responses in healthy individuals within an actionable time frame, offering valuable insights into personalized aging trajectories and early intervention strategies.

### Organ‐resolved features track lipid metabolism in aging‐related diseases

While our study identified significant commonality across organ communities, we were particularly motivated to investigate organ‐specific proteomic changes associated with aging. To achieve this, we calculated tissue specificity (TS) scores for each protein in individual tissues using the AdaTiss algorithm. We then applied a standardized TS score threshold to classify proteins as either organ‐specific, and organ‐enriched (collectively referred to as “organ‐resolved” proteins). Specifically, proteins with a TS score ≥ 4 in one tissue and at least 1.5‐fold higher than in any other tissue were defined as organ‐specific, while those with a TS score between 2.5 and 4 were considered as organ‐enriched if they did not meet the organ‐specific criteria.

**Figure 4:**
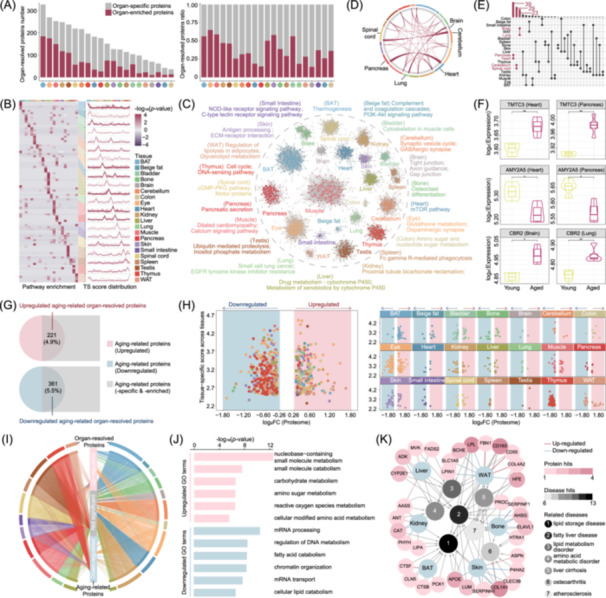
Organ‐resolved proteomic change and disease‐associated clues. (A) Identification of organ‐specific and organ‐enriched proteins in each tissue using AdaTiss algorithm. The left bar plot indicates their number and the right indicates proportion. (B) Heatmap of organ‐specific enriched pathways based on organ‐resolved proteins (left). Representative tissue specificity (TS) score distribution across different organs was demonstrated (right). Each line represents one protein. (C) The top pathways enriched in organ‐resolved proteins are shown for each organ. (D) The interorgan shared organ‐resolved proteins are shown in the circus plot. (E) The number of organ‐resolved proteins that are shared by more than one organ, with heart & pancreas, brain & lung and cerebellum & spinal cord encompassing the largest shared proteins and being highlighted. (F) Significant age differences of TMTC3 and AMY2A5 varying in heart & pancreas, as well as CBR2 varying in brain & lung. (G) Venn diagrams showing the overlapped proteins of aging‐related DEPs and organ‐resolved proteins. Bubble plots (H) and circular connection diagram (I) displaying organ distributions of aging‐upregulated or aging‐downregulated organ‐resolved proteins in all or separate tissue. (J) GO enriched terms of aging‐upregulated or aging‐downregulated organ‐resolved proteins. (K) Disease ontology enrichment results in aging‐related tissue‐resolved proteins of each tissue. The dark red circles indicate six involved organs, including liver, WAT, BAT, kidney, skin, and bone. The white‐to‐black legend indicates the number of proteins related to the indicated diseases, whereas the white‐to‐amber legend indicates the number of diseases related to the indicated proteins. The color of lines between the organ and proteins exhibits their corresponding expression changes.

Across the tissues analyzed, we identified 40–330 organ‐resolved proteins per tissue, accounting for 0.5%–8.3% of all detected proteins (Figure 4A). Notably, BAT displayed the highest count of both primary and tissue‐unique organ‐resolved proteins (Figure 4A, Figure S6A). The robustness and functional specificity of these proteins were validated through TS score distribution and KEGG pathway enrichment analyses (Figure 4B). Pathway enrichments for organ‐resolved proteins highlighted the core physiological functions of their respective organs (Figure 4C). For instance, thermogenesis in the BAT is essential for energy expenditure and heat production; tight junctions, axon guidance, and gap junction pathways in the brain are vital for neuronal communication and synaptic function. In addition, pathways crucial for immune response, host defense, and metabolism were also enriched, such as complement and coagulation cascades in the beige fat [30], the NOD‐like receptor and C‐type lectin receptor signaling pathways in the small intestine [31, 32], as well as amino sugar and nucleotide sugar metabolism in the colon [33]. The functional retention of organ‐resolved proteins facilitated our investigation into their specialized roles in aging and their potential connections to diseases.

We next sought to investigate organ‐organ crosstalk and shared molecular basis in tissue‐resolved aging. Upon comparison, we identified three major organ pairs: heart‐pancreas, cerebellum‐spinal cord, and brain‐lung, which shared 39, 29, and 23 organ‐resolved proteins, respectively (Figure 4D,E). Among these, several proteins exhibited dysregulated expressions during ageing, including TMTC3/AMY2A5/TRY5 in the heart‐pancreas and CBR2/GUCY1B1 in the brain‐lung (Figure 4F, Figure S6B–D). Notably, CBR2 functions as a crucial enzyme in fatty acid beta‐oxidation and exerts a protective role against oxidative stress, thereby influencing the aging process of high‐energy‐demand organs such as brain and lung, potentially alleviating the risk of neurodegenerative and pulmonary diseases. TMTC3 is vital for the endoplasmic reticulum and unfolded protein stress responses [34]. Targeting these pathways may preserve proteostasis, potentially slowing pancreatic islet β‐cell aging and preventing diabetes [35]. Dysregulated expressions of AMY2A5 and TRY5 were intricately associated with pancreatic digestive dysfunction and posed significant risks to cardiac health by exacerbating metabolic imbalance [36, 37]. GUCY1B1, which catalyzed the conversion of GTP to cGMP, might affect pulmonary function through cGMP‐related neural modulation [38].

Of all the organ‐resolved proteins examined, 221 were found to be upregulated in the aged group, representing 4.9% of the total, while 361 proteins were downregulated, making up 5.5% (Figure 4G and Table S2). Significant variations in the tissue distribution of differentially expressed organ‐resolved proteins were shown (Figure 4H,I). Significantly, the aged group was primarily associated with biomolecular metabolism pathways, including nucleobase‐containing small‐molecule metabolism, small‐molecule catabolism, and carbohydrate metabolism. In contrast, the young group showed enrichment in fatty acid catabolism and cellular lipid catabolism‐related pathways (Figure 4J). Taking these results, metabolic pathways, including lipid catabolism, exerted preeminent influences on aging‐related organ‐resolved effects characteristic.

To further clarify the relationships between organ‐specific aging and disease, a disease ontology enrichment analysis was conducted. Dysregulated organ‐resolved proteins from the liver, WAT, BAT, kidney, skin, and bone were enriched for several disease terms, suggesting a link between organ‐resolved aging and LMD‐related disorders, including lipid storage disease, fatty liver and liver cirrhosis (Figure 4K). These results suggest that these organs are more susceptible to aging‐related lipid metabolic reprogramming. Overall, this analysis underscores the intricate balance between organ‐shared and organ‐specific protein expression patterns, clarifies the molecular basis of organ crosstalk, and establishes a connection between aging and LMD disorders.

### Effect of aging on the metabolome of different tissues

**Figure 5:**
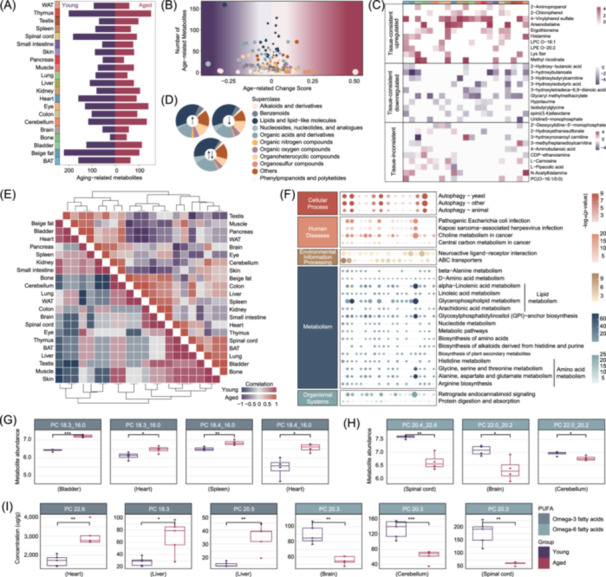
Identification of aging‐related metabolites varying in tissues. (A) Bar plots displaying the count of differential metabolites between the young and aged tissues. Aging‐accumulation labeled in the dark red, and aging‐reduction labeled in deep purple. (B) Aging‐related change score of different metabolite classes in each tissue. The size of the circles represents the absolute value of the score. (C) Tissue‐consistent upregulation and downregulation, as well as tissue‐inconsistent dysregulation of aging‐related metabolites across tissues. (D) The percentages of separate metabolite superclass compositions are shown in the pie charts. ↑, tissue‐consistent upregulation; ↓, tissue‐consistent downregulation; ↑↓, tissue‐inconsistent dysregulation. (E) Inter‐tissue correlation heatmaps displaying the metabolic coupling in the young (bottom left) and aged (top right) mice. (F) KEGG pathway enrichment analysis of aging‐related metabolites in various tissues. The aged group showed enhanced pathways in lipid and amino acid metabolism, autophagy, etc. Significant age‐related differences of omega‐3 (G) and omega‐6 (H) fatty acids that vary in specific tissues via untargeted metabolomics. (I) Concentration (μg/g) of omega‐3 and omega‐6 fatty acids in the young and aged heart, liver, brain, cerebellum and spinal cord measured by targeted metabolomics (*n* = 5 mice per group). PUFA, polyunsaturated fatty acids.

Following proteomic findings, we next examined the cross‐tissue metabolome to assess how aging influences metabolite profiles. We observed that metabolite differences were highly tissue‐dependent, reflecting system‐wide heterogeneity and variation by metabolite class (Figure 5A, Figure S7A). In adipose tissues, BAT and beige fat shared similar aging‐related metabolites quantities and class compositions, whereas WAT showed distinct metabolic patterns. Similarly, in the nervous system, the brain and cerebellum had more comparable metabolite profiles, which significantly differed from those in the spinal cord (Figure S7A).

An aging‐related metabolite change score revealed unique alterations in each organ. For example, alkaloids and derivatives decreased in the pancreas, heart, and eye, while organosulfur compounds accumulated in the heart and spinal cord (Figure 5B, Figure S7B). We further evaluated how these aging‐related metabolite changes overlapped among tissues (Figure 5C,D). Tissue‐consistent upregulated metabolites were mainly linked to histidine, cholesterol, and glycerolipid metabolism, whereas tissue‐consistent downregulated metabolites were enriched in the biosynthesis of cofactors, glutathione metabolism and longevity‐regulating pathways (Figure S7C).

Since synchronized inter‐tissue regulation of metabolic pathways is crucial for maintaining homeostasis, we used a Spearman correlation heatmap to illustrate metabolic coupling among 21 tissues in both young and aged mice [39] (Figure 5E). Our analysis focused on changes within the three abovementioned organ communities, including Cluster 1, Cluster 2 and the LMD disorder‐related organs (Figure 2F, Figure 4K). In Cluster 1, the correlations among the colon, small intestine, and spleen intensified in aged mice, while the thymus became increasingly distant from these tissues. Within Cluster 2, the most prominent changes were the evolving linkage of the brain and a complete reversion of WAT. Moreover, assessing the six LMD disorder‐related organs revealed a diminished correlation network among the aged liver, BAT, bone and skin (Figure S7D).

Pathway enrichment analysis of differential metabolites showed that the aged group elicited a robust enrichment in lipid metabolism, amino acid metabolism, and autophagy pathways (Figure 5F). Alpha‐linolenic acid, linoleic acid, arachidonic acid and glycerophospholipid metabolism, which are vital in inflammatory responses regulation and overall metabolic balance, were among the primary lipid metabolism processes affected [40]. These findings promoted us to examine key fatty acids underlying these metabolic activities. We observed omega‐3 fatty acids (PC 18.3_16.0, PC 18.4_16.0) were abundant in the heart, bladder and spleen (Figure 5G), while omega‐6 fatty acids (PC 20.4_22.6, PC 22.0_20.2) were reduced in the nervous system (Figure 5H). The altered levels of omega‐3 and omega‐6 polyunsaturated fatty acids (PUFAs) in cardiovascular or nervous systems may relate to inflammatory stress response during aging [41, 42]. Previous studies indicate that PUFAs play complex roles in the aged liver, which undergoes multifaceted senescence‐related challenges such as cell death‐related inflammatory injury [43], innate immune responses [44] and circadian rhythm‐related metabolic dysfunction [45]. To confirm these findings, we performed ex vivo targeted metabolomics on young and aged heart, liver and nervous system tissues. Consistent with our global metabolomic data, omega‐3 fatty acids accumulated in the aged heart and liver, while omega‐6 fatty acids uniformly reduced in the aged brain, cerebellum and spinal cord (Figure 5I). Overall, our results indicate that the critical alteration in unsaturated fatty acid metabolism may serve as a key metabolic hub in aging.

### Functional dysbiosis in the gut microbiota was associated with fatty acid metabolic remodeling during aging

**Figure 6:**
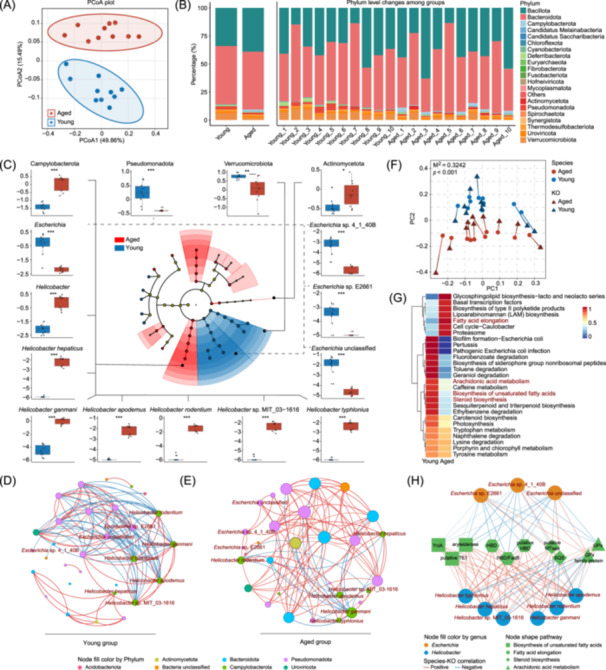
Remarkable changes of the gut microbiota symbiotic networks and functions in the aged mice. (A) Principal coordinate analysis (PCoA) with Bray–Curtis dissimilarity based on the species relative abundances in the young and aged groups (*n* = 10 mice per group). (B) Component proportions of the microbes at the phylum level in the young and aged groups (left) or samples (right). (C) Cladogram illustrating statistical differences supervised by Linear discriminant analysis effect size (LEfSe) method. Age‐related differences of critical gut microbial abundance, including differential phyla, *Escherichia spp*. and *Helicobacter spp*., were shown in boxplots. Separate correlation network of the top 30 differentially abundant gut microbes in the young (D) or aged (E) groups. Node color indicates the belonging phylum communities of species. The dark red font indicates critical bacteria. The positive or negative correlations are presented in red or blue lines, respectively (Spearman, |rho| > 0.6, *p* < 0.05). (F) Procrustes analysis to assess the correlation between microbial community and function, based on PCoA (Bray–Curtis) results of species abundances and KEGG orthologs (KOs) in young and aged groups. (G) Significant altered metabolic pathways in the gut microbial profiles of aged mice. The color bar denotes the normalized total transcripts per million (TPM) for genes belonging to this pathway. Significant dysregulated pathways are labeled with red fonts. (H) Species‐KO correlation network between key aging‐related intestinal bacteria and KO genes. GPx, glutathione peroxidase; HBD, 3‐hydroxybutyryl‐CoA dehydrogenase; MTase, methyltransferase; SQS, Squalene/phytoene synthase; TE1, acyl‐CoA thioesterase i protein.

Growing evidence indicates that alterations in the composition and function of the gut microbiota play a central role in immunosenescence, as well as inflammatory and metabolic aging processes [46]. To investigate this, we performed shotgun metagenomic sequencing on fecal samples from young and aged mice (*n* = 10 mice per group). The average sequencing depth was sufficient for exploring gut microbiomes and did not differ significantly between the two groups (Figure S8A). While the aged group showed a slight but nonsignificant increase in the Shannon and Chao1 diversity indices (Figure S8B,C), both principal coordinate analysis and nonmetric multidimensional scaling confirmed a significant separation between young and aged microbiomes (Figure 6A, Figure S8D).

At the phylum level, Campylobacterota and Actinomycetota were dramatically increased in aged mice, whereas Verrucomicrobiota and Pseudomonadota were significantly decreased (Figure 6B, Figure S8E). At the genus level, decreases in *Escherichia* and an increase in *Helicobacter* were the most prominent changes (Figure S8F). Similarly, at the species level, several potentially pathogenic bacteria, such as *Campylobacter* sp. RM8970, *Helicobacter apodemus* and *Helicobacter hepaticus*, were significantly increased in aged mice (Figure S8G). Linear discriminant analysis effect size (LEfSe) analysis confirmed these shifts, showing greater abundance of three *Escherichia* species in the young group (*Escherichia* sp. 4_1_40B, sp. E2661, and unclassified) and six *Helicobacter* species in the aged group (*Helicobacter apodemus*, *ganmani*, *hepaticus*, *rodentium*, sp. MIT_03‐1616, and *typhlonius*) (Figure 6C, Figure S9A).

Since the gut microbiota is governed by complex symbiotic or antagonistic interactions, any disruption can profoundly influence pathophysiological processes. In our study, *Escherichia spp*. showed a marked decline in aged mice, whereas *Helicobacter spp*. became more abundant. Notably, these shifts were negatively correlated, suggesting competitive or antagonistic relationships (Figure S9B). In young mice, the intestinal abundances of three *Escherichia* species (*Escherichia* sp. 4_1_40B, sp. E2661, and unclassified) were strongly inversely correlated to all six *Helicobacter* species mentioned above. However, these relationships were no longer present in aged mice (Figure 6D,E).

To clarify how gut microbiota dysbiosis might contribute to aging, we assembled the metagenomic reads into contigs, identified nonredundant genes (Figure S9C,D), and performed functional annotation using the KEGG database. Our analysis identified a strong correlation between microbial species and their functional profiles, indicating that microbiome composition can predict microbiome function (Figure 6F). In aged mice, pathways related to arachidonic acid metabolism, the biosynthesis of unsaturated fatty acids and steroid biosynthesis declined, while fatty acid elongation process increased (Figure 6G). Several genes encoding key enzymes in fatty acid metabolism also showed difference in the aged mice, such as HADH (K00022), SMT1 (K00559) and gpx (K00432) (Figure S9E). Moreover, the correlation network demonstrated that the three *Escherichia* species and six *Helicobacter* species had opposing correlations with KEGG orthology (KO) genes involved in lipid metabolism, suggesting their opposite regulatory roles in aging‐related lipid metabolic reprogramming (Figure 6H).

Taking together, these findings signified the dysbiosis of gut microbiota in aging mice, characterized by the competitive or antagonistic interaction between *Escherichia* and *Helicobacter*, is closely related to lipid metabolism, particularly involving unsaturated fatty acids.

### Integrative omics analysis and molecular hub interpretation

**Figure 7:**
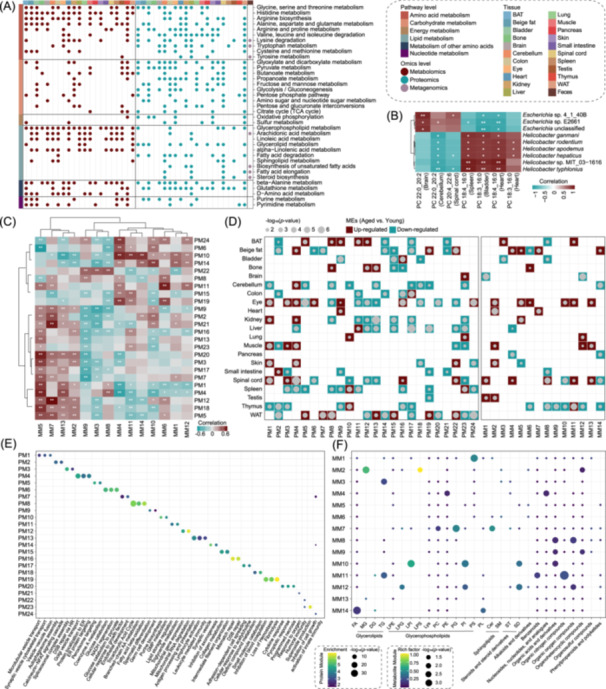
Integrative omics‐resolved alterations to multiorgan aging. (A) Significant enrichments for relevant categories of KEGG metabolic pathways from features that were dysregulated in various organs detected by proteome, metabolome and metagenome. (B) Notable correlations of the abundances between dysbiotic fecal species of *Escherichia*/*Helicobacter* and tissue‐derived omega fatty acids. (C) Heatmap of Spearman correlations between the module eigenfeatures (MEs) of proteomics module 1‐24 (PM1‐24) and metabolomics module 1‐14 (MM1‐14). Statistical significance was determined by two‐sided Student's *t*‐tests of the transformed correlations. (D) Aging‐related changes of the levels of proteomics and metabolomics MEs across different tissues. Upregulation is indicated in red, while downregulation is shown in bluish green. (E) The KEGG enrichment analysis of separate weighted gene coexpression network analysis (WGCNA) proteomic modules. (F) The metabolite enrichment analysis of separate WGCNA metabolomic modules.

Drawing from the analysis above, we were prompted to investigate the overall metabolic restructuring associated with aging across multiple tissues. Perturbed pathways enriched in integrative omics datasets, including proteome, metabolome, and fecal metagenome were analyzed. As a result, aging had broad regulatory effects on a variety of biomolecule metabolisms (Figure 7A). Notably, gut microbiota dysbiosis‐related alterations exclusively related to lipid or amino acid metabolism. Integrative omics enrichments for dysbiosis‐related fatty acid metabolic remodeling were found abundant in the liver, cerebellum and spinal cord. Correlation analysis between aging‐related differential microbes and PUFAs of interest was then carried out (Figure 7B). The results showed that of *Escherichia* and *Helicobacter* exerted opposing effects on omega‐3 and omega‐6 fatty acids across multiple tissues.

Next, we used weighted gene coexpression network analysis (WGCNA) algorithm to generate modules, defined as groups of highly correlated features in proteomic and metabolomic profiles, and assessed correlations between inter‐omics module eigenfeatures (MEs) (Figure 7C). Tissue abundances, functional pathway enrichment and metabolite classification were performed to characterize each module (Figures S10–12). Proteomics modules were denoted by PM, while metabolomics modules were denoted by MM. The ME of the largest proteomics module, PM1, was positively correlated with MM2, both of which were increased in the eye, muscle and skin during aging (Figure 7C,D). PM1 was primarily associated with the vesicle export and recycling processes, which may regulate the transportation and secretion of MM2‐enriched MG, LPS and organoheterocyclic compounds during aging (Figure 7E,F). The second largest proteomics module, PM2, showed enrichment in autophagosome assembly and the calcineurin‐NFAT signaling cascade, with a uniformly lower ME in adipose tissues (BAT, WAT, and beige fat) and a significant correlation with PC/PG/Cer‐abundant MM7 (Figure 7C–F). These observations suggest that impaired adipose tissue homeostasis in glycerophospholipids and sphingolipids may increase susceptibility to obesity and other metabolic diseases with aging. Additionally, two other modules involving lipid metabolism were identified. PM8 exhibited high enrichments in fatty acid beta‐oxidation and tricarboxylic acid cycle, while PM11 featured by lipid storage regulation and triglyceride (TG) metabolism (Figure 7E). The MEs of PM8 and PM11 were elevated in several aged tissues and closely associated with PC‐rich MM7, suggesting a link between loss of proteostasis and fatty acid metabolic remodeling during aging (Figure 7C–F).

Remarkably, we also identified pronounced organ‐resolved and organ‐shared effects on proteome‐metabolome relationships (Figure 7C–F). In the aged kidney, PM4 and MM5 were closely linked, with PM4 exhibiting strong enrichment in endoplasmic reticulum stress responses, pointing to its potential significance in regulating metabolic activity during renal aging. In contrast, PM10, related to GMP biosynthesis, declined in the aged nervous system and was significantly correlated with PE/nucleotide‐rich MM4, suggesting that in sufficient GMP supply may impair fatty acid oxidation and organic nitrogen metabolism. Moreover, the level of PM16, characterized by DNA damage repair deficiency, and the TG‐containing MM11 changed simultaneously in the aged thymus, exhibiting a strong correlation that aligns with prior enrichment analyses (Figure 2B). This finding underscores the significance of DNA damage repair in preventing LMD during aging. Intriguingly, we also identified PM12 as an organ‐shared module altered in multiple LMD disorder‐related organs, including liver, BAT, WAT, bone and kidney. PM12 was primarily involved in mitochondrial RNA degradation and was closely associated with PC‐abundant MM7, indicating the link between aging‐related mitochondrial dysfunction across tissues and LMD disorders.

As the close association between PM2, PM8, PM11, PM12, and MM7 emerged, implicating a synergistic aging process in adipose tissue (BAT, WAT and beige fat), liver, kidney and bone, we sought to explore protein‐metabolite interactions within these modules (Figure S13). Interestingly, omega‐3 fatty acids (PC 18:1_22:6) and omega‐6 fatty acids derivatives (LPC 20:4) within MM7 were identified as the central metabolites participating in protein‐metabolite interactions. Significant interplay was observed between omega fatty acids and components of the mitochondrial trifunctional protein (TFP) complex, specifically LPC 20:4 versus HADHA, and PC 18:1_22:6 versus HADHB (Figure S13). By modulating PUFA catabolism, HADHB and HADHA play a pivotal role in maintaining cellular energy balance and reducing oxidative stress. The results demonstrate how interactions between omega fatty acids and HADHA/HADHB occur across multiple tissues, highlighting the significance of mitochondrial oxidation for omega fatty acids in the coordinated aging process.

## DISCUSSION

With the emergence of a multiorgan aging network, understanding the organ‐resolved changes is essential for gaining a comprehensive view of healthy aging [47, 48, 49]. In this study, we employed an integrated‐omics approach to thoroughly investigate the key molecular hub governing interactions among proteins, metabolites, and gut microbiota, along with the crosstalk between circulating and parenchymal aging in mice. Utilizing omics data from diverse tissues, we revealed multidimensional proteomic alterations in tissue‐shared, ‐specific, ‐enriched, and ‐universal patterns, primarily centered on chronic inflammation and lipid metabolic remodeling. In aged mice, immunoglobulin accumulation in most tissues coincided with classical complement system activation in circulation, indicating a synergistic inflammatory process. Furthermore, tissue‐resolved proteomic changes suggested LMD, consistent with disrupted fatty acid metabolism driven‐by gut microbiota dysbiosis and age‐dependent changes in omega‐3/‐6 fatty acids. Collectively, our study outline the aging‐dependent proteomic‐metabolomic‐microbial landscape from a cross‐tissue perspective, highlighting systemic chronic inflammation and dysbiosis‐related fatty acid remodeling as major contributors to the aging process.

Inflammaging is a chronic, low‐grade inflammatory state associated with aging that has emerged as a critical driver of age‐related diseases, including metabolic disorders and cardiovascular diseases [16, 50, 51]. Contributing factors include the senescence‐associated secretory phenotype produced by accumulated senescent cells and the buildup of damage‐associated molecular patterns [52]. Our study builds on these findings by showing a significant immunoglobulin accumulation in aged tissues, consistent with recent spatial transcriptomic analyses of aging mammalian tissues [1]. This broadens our understanding of immunoglobulin‐associated senescence and underscores its systemic impact. Immunoglobulin accumulation may promote inflammaging through multiple mechanisms. For instance, macrophage recycling triggered by neonatal Fc receptor (FcRn) can exacerbate metabolic decline in aging WAT [53], while pro‐aging N‐glycans on IgG can comprise Fc‐mediated immune function in chronic HIV infection [54]. Further research could explore immune system dysfunction (e.g., antigen cross‐presentation, B cell alteration), decreased clearance (e.g., decline in reticuloendothelial system function, autophagy‐lysosomal dysfunction), oxidative stress and gut dysbiosis. Potential gero‐protective strategies aimed at regulating immunoglobulin dynamics include senolytics, FcRn inhibitors and anti‐CD20 Rituximab. However, careful optimization is necessary, as uncontrolled IgG depletion raises the risk of systemic infections.

IgG is one of the most abundant antibodies in the body and functions through mechanisms such as neutralization, opsonization, antibody‐dependent cell‐mediated cytotoxicity, and complement system activation. However, the specific target and actionable pathways by which immunoglobulins drive inflammaging remains unknown. By integrating plasma proteomics data, we identified classical complement system activation, possibly triggered by antigen‐antibody complex, and a synergistic enhancement of both innate and humoral immune responses in plasma and tissues. An increase in C4b with aging was shown to delay muscle progenitor cell proliferation and impair functional recovery, implying that C4b inhibition may improve muscle repair in older adults [55]. Nevertheless, we observed that complement proteins elevations were more pronounced in the aged circulation than in multiple aging tissues. Earlier work indicates that neurovascular decline can induce vascular leakage, linking C1QA upregulation in microglia/monocytesand to heightened neuroinflammation [56]. This finding suggests that tissue barrier dysfunction and increased vascular permeability may be crucial for translating circulating complement activity into localized tissue inflammation. Moreover, emerging evidence supports the potential of circulating complement proteins as biomarkers for biological aging and aging‐related diseases. For instance, increased serum C1q strongly correlates with TNF‐α or IL‐6 and reflects increased arterial stiffness in older populations [57]. Here, our longitudinal analysis of healthy individuals showed strong correlations among complement protein level, inflammatory factors and actionable‐time aging. Identifying complement proteins as novel biomarkers could thus yield valuable insights into individualized aging trajectories, enabling more personalized management and earlier interventions. Nonetheless, a multi‐center cohort study and well‐designed clinical trial for tracking circulating plasma complement proteins are required to better clarify their impacts on actionable‐time aging.

Proper nutritional composition and metabolic conditions are essential for maintaining normal immune function and alleviating inflammaging [58]. Our integrative proteomic‐metabolomic findings reveal a remarkable correlation between aging‐related mitochondrial decline and fatty acid metabolism. This relationship may promote LMD disorders through impaired fatty acid oxidation, increased production of reactive oxygen species (ROS), altered lipid droplet dynamics, and dysregulated lipid homeostasis‐related pathways, such as AMPK and PPAR. Although various biomolecule metabolic processes are crucial for age‐related metabolic reprogramming [59], most studies focus on a single organ and fail to capture broader, organ‐shared changes. Here, our cross‐tissue untargeted metabolomics analysis reveals the significant role of PUFA metabolism in aging. We identified substantial alterations in omega‐3 and omega‐6 fatty acids in the aged heart, liver, and nervous systems. These changes may be explained by reduced metabolic rate and fat accumulation [60], compensatory anti‐inflammatory responses to maintain membrane function [61], and aging‐related alterations in enzyme activity [62]. Current research on omega fatty acids levels in aging populations is inconsistent, partly due to variations in study design, participant demographics, and analytical methods. Furthermore, having a higher baseline omega‐3 level in older adults does not necessarily predict the therapeutic potential of omega‐3 supplementation. This highlights the need for further exploration into the diverse benefits of omega‐3 treatment for older adults with different baseline omega‐3 levels. Nonetheless, large clinical trials have demonstrated that omega‐3 fatty acids treatment (1 g per day) slows DNA methylation clocks, amplifying the gero‐protective effect of vitamin D and exercise [63]. Similarly, an omega‐3‐alone regimen reduced infection rate by 13% [64]. These findings suggest a need to explore the molecular interactions between immunoglobulin‐complement augmentation and epigenetic regulation under omega‐3 treatment. Given the diverse effects of omega‐3 and omega‐6 fatty acids on inflammation, their ratio in aging individuals significantly impacts the process of inflammaging. Optimizing this ratio through dietary interventions, disease prevention, personalized nutrition, and drug development holds important application value.

Herein, the marked decrease of *Escherichia* and increase of *Helicobacter* were found in the aged mice, indicating gut microbiota dysbiosis. *Escherichia* abundance has been linked to longevity in extremely long‐lived individuals (94–105 years old) [65, 66]. As for *Helicobacter*, fewer studies were conducted to investigate its function in aging. One study revealed that *Helicobacter* promotes gastric epithelial cell senescence that depended on CXCR2 signaling [67], but the specific molecular mechanisms by which these microbes regulate host metabolism remain unclear. Recent studies suggest that *Escherichia* and *Helicobacter* may influence host metabolism by producing bacterial metabolites, such as SCFAs and secondary bile acids, which modulate host enzymes like Δ‐6 desaturase and PPARs [68, 69]. For instance, butyrate, a key SCFA produced by *Escherichia*, has been shown to enhance fatty acid oxidation and mitochondrial function, while *Helicobacter* may disrupt these processes by inducing chronic inflammation and oxidative stress via NF‐κB and MAPK pathways [70, 71]. Additionally, *Helicobacter* infection has been shown to generate ROS and reactive nitrogen species (RNS) through the activity of its virulence factors, such as CagA and VacA [72], which disrupt mitochondrial function, impair antioxidant defenses, and cause oxidative damage to biomolecules. Our findings also highlights the significant value of mitochondrial activity‐related metabolism during aging. Dysregulated ENTPD1/ENTPD2/ENTPD4 and AK2/AK4 are crucial to purine metabolism; additional alterations include PC metabolism‐related mitochondrial RNA degrading process and omega fatty acid metabolism‐related mitochondrial TFP complex. These findings prompt speculation that *Escherichia* and *Helicobacter* might influence aging‐related PUFA metabolism through mitochondrial activity, which requires further validation. In addition, *Helicobacter* may alter the gut microbiota composition by reducing beneficial, SCFA‐producing bacteria, thereby exacerbating systemic inflammation and metabolic dysfunction. Collectively, these mechanisms create a pro‐inflammatory and pro‐oxidative microenvironment that accelerates cellular senescence and tissue damage, contributing to metabolic aging and age‐related diseases. Owing to the complexity of host‐microbe interactions, continued research is essential to fully elucidate these processes.

By combining shotgun metagenomic sequencing and untargeted metabolomics, we uncovered a strong correlation between the dysbiosis of *Escherichia* and *Helicobacter* and disrupted omega‐3 and omega‐6 fatty acid metabolism. Gut microbiota dysbiosis significantly influences fatty acid metabolism by modulating dietary fatty acid processing (e.g., converting fiber to SCFAs) and regulating host enzymes (e.g., Δ‐6 desaturase), thereby altering the omega‐6/omega‐3 ratio. An elevated omega‐6/omega‐3 ratio promotes pro‐inflammatory mediators, exacerbating chronic inflammation, while omega‐3s generate anti‐inflammatory mediators (e.g., resolvins) to counteract inflammation. Chronic inflammation, in turn, disrupts the gut microbial ecosystem, creating a vicious cycle. Complement component C3 directly interacts with gut microbiota, increasing intestinal permeability and promoting systemic inflammation [73]. These findings underscore the critical crosstalk between inflammation and microbiota in the aging process and highlight how this interplay can contribute to age‐related diseases. However, many aspects of this relationship remain to be elucidated. For instance, the precise mechanisms by which microbial‐derived metabolites like SCFAs and secondary bile acids regulate key metabolic pathways in different tissues (e.g., liver, adipose tissue, and brain) remain under investigation. Recent studies have demonstrated that SCFAs can modulate host metabolism through GPCRs and HDAC inhibition, leading to tissue‐specific effects on energy balance and inflammation [74].

Individual differences, such as genetic background, diet, and lifestyle, significantly influence the composition and function of the gut microbiota, as well as host metabolic responses. For instance, polymorphisms in genes encoding fatty acid desaturases (FADS1 and FADS2) have been linked to variations in omega‐3 and omega‐6 metabolism, which may explain interindividual differences in inflammatory responses and disease susceptibility [75]. Additionally, body weight and dietary habits could alter the gut microbiota composition, further modulating host metabolism. For example, high‐fat diets have been shown to reduce the abundance of *Bifidobacterium* and *Lactobacillus*, leading to increased intestinal permeability and systemic inflammation [76]. These findings underscore the importance of personalized approaches in metabolic aging research, considering genetic, dietary, and lifestyle factors. Although mice and humans share many similarities in gut microbiota composition and function, there are also distinct differences. For instance, the relative abundance of *Bacteroides* and *Firmicutes*, varies between two species [77]. Additionally, the metabolic rates and lifespans of mice differ greatly from those of humans, potentially affecting the translation of findings. Future work should focus on validating these findings in human cohorts, particularly in elderly populations, to clarify the role of gut microbiota in human aging. Longitudinal studies tracking changes in gut microbiota composition and metabolic profiles over time could provide insights into the causal links between dysbiosis and age‐related metabolic dysfunction.

Overall, our findings reveal an inseparable association among chronic inflammation, fatty acid metabolic dysfunction, and gut microbiota dysbiosis in aging, highlighting their collective impact on age‐related decline. Targeting these interconnected pathways through dietary, microbial, or therapeutic interventions offers significant potential to counteract aging‐related pathologies and promote the rejuvenation of inflammatory, metabolic, and microbial health.

## CONCLUSION

Our study presents a comprehensive proteomic‐metabolomic‐metagenomic profile of aging, spanning multiple tissues and chronological stages. Importantly, we identified the synergistic activation of the circulating complement system and parenchymal tissue‐universal immunoglobulin as essential molecular drivers of inflammaging. This synergy also contributed to gut microbiota dysbiosis‐mediated lipid metabolic reprogramming, particularly in PUFA metabolism, linked to the decline in *Escherichia* and the rise in *Helicobacter*. Elucidating the regulatory interplay between inflammaging and dysbiosis‐related fatty acid metabolic remodeling across different tissues in mammalian aging holds significant potential for discovering gero‐protective targets and pathways that can mitigate aging‐related diseases and promote overall health span.

## METHODS

### Mouse model and biological sample collection

C57BL/6 male mice, either 2 months or 20 months old (referred to as young or aged groups), were obtained from the Model Animal Research Center of Nanjing University. All mice were maintained in a controlled setting characterized by a 12‐h light/dark cycle, with the temperature kept at 20–25°C and humidity at 30%–70%. The mice were housed together for 7 days before experiments and provided with a standard chow diet and free access to drinking water, under a specific pathogen‐free environment throughout the study. The animal experiments were conducted in accordance with Chinese legislation governing the use of experimental animals and received approval from the Institutional Animal Care and Use Committee (IACUC) at Jennio Biotech Co., Ltd. (Approval No. JENNIO‐IACUC‐2024‐A041).

Before tissue dissection, fresh cecum fecal samples (100 mg per mouse) were collected under sterile conditions. After anesthesia, blood was acquired through cardiac puncture before transcardial perfusion with 20 mL PBS. EDTA‐plasma was then isolated by centrifugation at 1200 *g* for 10 min at 4°C. Subsequently, a variety of tissues—including heart, lung, liver, colon, small intestine, pancreas, bladder, spleen, kidney, testis, thymus, bone, muscle, whole brain, spinal cord, cerebellum, BAT, WAT, beige fat, skin, and eye—were collected in duplicate for proteomic and metabolomic sequencing from randomly selected mice across both young and aged groups. Following collection, the tissues, plasma and fecal samples were promptly snap‐frozen on liquid nitrogen and stored at −80°C.

### Sample preparation for proteomic analysis

Collected tissues were lysed with steel balls, lysis solution (containing 8 M Urea/50 mM Tris‐HCl), and Roche cocktail (1X). The crude lysates were placed on ice for 5 min, homogenized using tissue lyser (60 Hz, 2 min) and centrifuged at 20,000 *g* for 15 min at 4°C. The extracted proteins were reduced with 10 mM DTT in 37°C water bath for 1 h and then alkylated with 20 mM IAA at room temperature for 30 min, protected from light. The supernatants were collected in a new tube, followed by the determination of protein concentration via quantitative Bradford method. Protein samples were digested with trypsin (protein‐to‐enzyme ratio = 50:1) and incubated at 37°C for 16 h. The enzymatically digested peptides were desalted using waters solid phase extraction cartridges, vacuum dried, redissolved, and stored at −20°C.

A TMT‐based strategy was applied for proteomic analyses of parenchymal tissue samples. A proper amount of vacuum‐dried peptide from each sample was redissolved in 30 microliter (μL) of 100 mM TEAB, then reacted with each dissolved TMT‐10plex label at a TMT label‐to‐peptide ratio of 2:3 for 1–2 h at room temperature. To improve the depth of protein identification, equal amounts of peptide from each sample were mixed, diluted with solvent A (5% ACN, pH = 9.8) before being injected into a 3.5 micrometer (μm), 4.6 mm × 150 mm Agilent ZORBAX 300Extend‐C18 column (for plasma samples), or a 3.0 μm, 2.0 mm × 150 mm Phenomenex Gemini NX‐C18 column (for tissue samples) on a Thermo Scientific UltiMate™ 3000 Binary Rapid Separation System. The gradient elution was performed at a flow rate of 0.25 ml/minute, progressing from 5% to 21% solvent B (97% ACN, pH = 9.8) over 38 min, followed by an increase to 40% in 20 min, a quick rise to 90% in 2 min, maintenance at 90% for 3 min, and finally a re‐equilibration at 5% for 10 min. Elution peaks were monitored at 214 nm, and fractions were collected every minute, leading to 10–15 fractions that were freeze‐dried.

### LC‐MS/MS analysis and protein identification

The desalted peptide samples were reconstituted in a solution of 0.1% formic acid and then centrifuged at 20,000 *g* for 10 min. The resulting supernatant was collected and loaded into a self‐packed C18 column (particle size, 1.8 μm; pore size, 100 Å; length 35 cm. Thermo Fisher Scientific).

For tissue samples, proteomic analyses were performed by Thermo Scientific UltiMate 3000 coupled with QE HF‐X™ system. Samples were injected into the trap column (Acclaim PepMap 100, 75 μm × 20 mm, C18), and separated on the analytical column (75 μm × 250 mm, C18) using a gradient over 110 min with solvent A (0.1% formic acid) and solvent B (80% ACN, 0.1% formic acid) at a flow rate of 300 nL/minute. The Parameter settings were as below: MS1 at a resolution of 60,000, AGC target 3E6 charges, scan range 350–1800 m/z; MS2 at a resolution of 45,000, AGC target 2E5 charges, max IT was 105 ms.

For plasma samples, proteomic analyses were performed by Thermo Scientific EASY‐nLC™ 1200 coupled with Orbitrap Exploris™ 480 system. Separation was performed at a 300 nL/min flow rate through the following effective gradient: From 0 to 103 min, 4% solvent B (98% ACN, 0.1% formic acid) was linearly increased to 27%; 103–111 min, solvent B ranging from 27% to 40%; 111–113 min, solvent B ranging from 40% to 90%; 113–120 min, 90% solvent B. The Parameter settings were as below: MS1 at a resolution of 60,000, AGC target 3E6 charges, scan range 350–1500 m/z; MS2 at a resolution of 15,000, AGC target standard charges, max IT was 22 ms.

The raw data from LC‐MS/MS analysis was processed using MaxQuant (v2.1.4.0). The mass tolerances for precursor peptides and fragment ions were configured to 20 ppm and 0.05 Da, respectively. Cysteine carbamidomethylation was designated as a fixed modification, while methionine oxidation and N‐terminal acetylation of proteins were assigned as variable modifications. The LC‐MS/MS data were searched against protein sequences obtained from the UniProt database. Reliable unique peptides were defined as peptides uniquely mapped to a protein with FDR ≤ 1%, supported by ≥2 unique peptides, length ≥7 amino acids, mass accuracy ≤20 ppm, and Andromeda score ≥40.

### Untargeted metabolomic analysis

The collected samples were thawed on ice, and hydrophilic metabolites were extracted with 80% (v/v) methanol buffer by adding 1 mL methanol to 50 mg of tissue. The extraction mixture was transferred into a new tube, after which the metabolites were fully extracted at −20°C for 30 min. After centrifugation at 20,000 *g* for 15 min, the supernatants were collected and vacuum dried. The samples were redissolved with 50% acetonitrile and stored at −80°C before the LC‐MS analysis. In addition, 10 μL from each extraction were mixed and used as pooled QC samples for MS detection.

Thermo Scientific UltiMate 3000 coupled with QE HF‐X™ system were used to analyze the hydrophilic metabolites extracted from young and aged mice tissues. Accucore‐150‐Amide‐HILIC System (100 mm × 2.1 mm, 2.6 μm, Thermo Fisher Scientific) was used for the reversed‐phase separation. The column oven was kept at 40°C. In positive ion mode, gradient elution was performed using mobile phase A (50% ACN, 10 mM Ammonium formate, 0.1% formic acid) and mobile phase B (50% ACN, 10 mM ammonium formate); In negative ion mode, mobile phase A (50% ACN, 10 mM Ammonium formate) was used. Gradient elution conditions were set as follows: 0–0.5 min, 98% B; 0.5–5 min, 98% to 80% B; 5–11.3 min, 80% to 50% B; 11.3–15.3 min, 50% to 2% B; 15.3–17.3 min, 2% B; 17.3–17.4 min, 2% to 98% B; 17.4–20 min, 98% B.

A high‐resolution tandem mass spectrometer QE HF‐X™ (Thermo Fisher Scientific) was used to detect polar metabolites eluted from the column. The Q‐Exactive was operated in both positive and negative ion modes. Precursor spectra (70–1050 m/z) were collected at 70,000 resolution to hit an AGC target of 3e6. The maximum IT was set to 100 ms. A top 3 configuration to acquire data was set in DDA mode. Fragment spectra were collected at 17,500 resolution to hit an AGC target of 1e5 with a maximum IT of 80 ms.

The raw files of LC−MS were converted into mzXML format and then processed by the XCMS, CAMERA and metaX toolbox based on an in‐house fragment spectrum library of metabolites [78, 79, 80]. Each ion was identified by combining retention time (RT) and m/z data. Intensities of each peak were recorded, and a three‐dimensional matrix containing arbitrarily assigned peak indices (retention time‐m/z pairs), sample names (observations) and ion intensity information (variables) was generated. Metabolites that were detected in less than 80% of biological samples or with CV values ≥ 0.3 among the QC replicates were removed. Missing values were imputed with the k‐nearest neighbor algorithm to further improve the data quality.

### Targeted metabolomic analysis

Targeted metabolomics analysis was conducted using gas chromatography‐tandem mass spectrometry. Tissue samples (50 mg) were homogenized in a solution containing 200 μL methanol, 50 μL 36% phosphoric acid, 200 μL deionized water, and 200 μL methyl tert‐butyl ether. Following homogenization, the mixture was centrifuged, and the supernatant was extracted twice, pooled, and dried under nitrogen. Methyl esterification was carried out using 14% boron trifluoride‐methanol, after which 200 μL saturated NaCl and 500 μL hexane were added. The solution was vortexed and centrifuged at 20,000 rpm for 15 min, and the hexane layer was collected for analysis. Separation was performed on a TR‐FAME column (100 m × 0.25 mm, 0.2 μm) with helium as the carrier gas, an injection volume of 1 μL, and an inlet temperature of 250°C. Detection was achieved using a GCMS‐TQ8040 NX triple quadrupole mass spectrometer with an electron ionization (EI) source. EI parameters included an ion source temperature of 250°C, a transfer line temperature of 260°C, and a detection voltage of 0.8 kV. Quantification was performed using multiple reaction monitoring, with collision energy optimized for each target compound.

### Fecal DNA extraction and shotgun metagenomic sequencing

Genomic DNA was extracted from tissue samples using the Fecal Genome DNA Extraction Kit (AU46111‐96, BioTeke). DNA libraries were constructed via TruSeq Nano DNA Library Preparation Kit‐Set (FC‐121‐4001, Illumina) according to the manufacturer's instructions. Metagenome libraries were quantified using Qubit 1X dsDNA HS Assay Kits (Q33230, Invitrogen) and further sequenced on Illumina NovaSeq. 6000 platform with PE150. Sequencing adapters were removed from de‐multiplexed raw sequences using cutadapt [81]. Then, the low‐quality reads (quality scores < 20), short reads (<100 bp), and reads (>5% “N” records) were trimmed by sliding‐window algorithm method in fqtrim (v0.94, CNIC). Quality‐filtered reads were aligned to the mouse genome using bowtie to eliminate host contamination [82]. The remaining effective reads underwent de‐novo assembly using MEGAHIT (v1.2.9) for microbial function and taxonomy assignment [83]. Coding regions (CDS) of assembled contigs were predicted using MetaGeneMark [84], and CDS sequences were clustered into unigenes using CD‐HIT [85].

### Tissue specificity (TS) score for proteins

TS score for each protein in separate tissue was calculated using AdaTiss as previously described, thereby establishing robust metric that enables accurate identification of specified expression population [86]. Particularly, tissue‐specific protein was defined with TS score ≥4 in one tissue and was at least 1.5 higher than the protein's TS scores in any other tissues. When TS score was in the interval (2.5, 4) but the protein was not tissue‐specific, the protein was identified as tissue‐enriched [87, 88]. Tissue‐specific and ‐enriched proteins were collectively referred to as tissue‐resolved proteins (Conceptually equivalent to organ‐specific, organ‐enriched or organ‐resolved proteins), being used to investigate the effect of tissue specificity on aging.

### Pathway and disease ontology enrichment analysis

Pathway enrichment analyses were carried out based on Metascape Bioinformatics Resources [89], ClusterProfiler v4.0.0 [90] and OmicStudio tools (https://www.omicstudio.cn/tool) [91]. Disease ontology enrichment analysis was performed using the DOSE R package for aging‐related tissue‐resolved proteins of each tissue that had at least 5 proteins [92, 93]. Identified associations were declared as significant if *p* < 0.05 and they had at least 2 molecules in the intersection between input sets and the disease term sets. The software Cytoscape v3.9.1 was applied to visualize the functional and microbial networks [94].

### Correlation between plasma proteins and tissue proteins

Aiming to compare circulating protein changes with shifts in protein expression in any tissue, Spearman's rank correlation was calculated between a given protein simultaneously detected in plasma and at least one tissue [10]. The critical plasma‐tissue pair of certain proteins was screened out as followed: (1) The protein was captured by both plasma and tissue proteomic profiling (1183 proteins); (2) The protein in the plasma demonstrated significant change with age (135 proteins); (3) The protein in plasma and tissue pair expression profiles had to manifest a Spearman's rank correlation of more than 0.6; (4) The corresponding proteins had to be differentially expressed during aging in the certain correlated tissue (59 proteins; 94 plasma‐tissue pairs). As expected, a particular protein might exhibit correlations with expression changes across several tissues.

### Determination of aging‐related change score for metabolite subclasses

The aging‐related change score for metabolite subclasses across tissues was designed to assess the trend of changes in a group of features during aging, calculated using the formula: Aging‐related change score = (*N*_increase_ − *N*_decrease_)/*N*_total_. Accordingly, *N*_increase_, *N*_decrease_, and *N*_total_ represented the number of features that were increased, decreased, and the total features in a specific group. A positive score suggested that the feature group tended to accumulate with age, whereas a negative score indicated the reduction [95].

### Weighted gene coexpression network analysis (WGCNA)

WGCNA was conducted to summarize nonoverlapping modules of highly correlated features using intramodular hub features [96]. Herein, the modules were labeled as PM (for proteomic modules) and MM (for metabolomic modules) and were arranged in descending order by module size. A higher scale‐free topology fit index (*R*²), particularly above 0.90, indicates a closer alignment with the scale‐free model, highlighting the presence of highly connected hub nodes that are frequently essential in biological processes. In this study, soft thresholds of 12 for proteomic and 5 for metabolomic clustering were selected, corresponding to the minimum power values at which *R*² reaches 0.90. This ensures that the network approximates a scale‐free topology, consistent with the properties observed in many real biological networks. Additionally, the minimum module size was set to 30. Module eigengenes (MEs) were extracted as the first principal component and principal eigenvector, which can be considered representative of the expression or abundance profile within a module and used to calculate Spearman's rank correlation coefficients between modules [97, 98]. Two‐sided Student's *t*‐tests were applied to the transformed correlation coefficients to evaluate the significance.

### Microbial taxonomic and functional profiling

Taxonomic assessment of microbiota was performed with DIAMOND (v0.9.14) based on the NR database [99]. As a result, the profiles of microbial communities were established at various taxonomic levels, including kingdom, phylum, class, order, family, genus, and species. Linear discriminant analysis effect size (LEfSe, https://huttenhower.sph.harvard.edu/galaxy/) was employed to identify taxonomic features that exhibit differential abundance between the young and aged groups (LDA score > 3.0, *p* < 0.05). Microbial functions were determined using the KEGG database via DIAMOND (blastp, evalue ≤ 1e‐5) [100]. Each sequence was assigned to a KEGG orthology (KO) module based on the highest‐scoring annotated hits [101]. The abundance of each KO was calculated by summing the Transcripts Per Million for all genes associated with that specific feature. Additionally, the abundance of KEGG pathways was derived by aggregating the KO abundances corresponding to the same feature.

### Ex vivo validation

Immunofluorescence (IF) staining was performed according to the protocol described in our previous research [44]. Initially, tissue sections were subjected to incubation overnight at 4°C with primary antibodies targeting Rabbit anti‐mouse IgG (Abcam, Cat# 46540). Nuclei were stained with 4′,6‐diamidino‐2‐phenylindole (DAPI). ELISA were performed following the manufacturer's instructions. Commercial C2 (Cusabio, Cat# CSB‐EL003658MO), C4 (Elabscience, Cat# E‐EL‐M3020), C5a (Elabscience, Cat# E‐MSEL‐M0024), sC5b‐9 (Elabscience, Cat# E‐EL‐M1129) ELISA kits were used to measure their levels in plasma of young and aged mice.

### Statistical analysis

Statistical comparisons between two groups were conducted using either unpaired *t*‐tests or the Wilcoxon rank‐sum test, whereas differences among three or more groups were assessed using one‐way ANOVA or the Kruskal–Wallis test. Spearman's rank correlation analyses were performed to analyze the correlation between two sets of data. Bioinformatic analyses were performed by R v4.2.2 (http://www.R-project.org/). All statistical *p* values were two‐sided, with significance defined as *p < 0.05; **p < 0.01; ***p < 0.001; *ns*: not significant. Sample sizes are included in the figure legends.

## AUTHOR CONTRIBUTIONS

**Feng Zhang**: Conceptualization; writing—review and editing; project administration; writing—original draft; investigation; methodology; formal analysis; visualization; software. **Rong Li**: Writing—original draft; investigation; methodology; formal analysis; writing—review and editing. **Yasong Liu**: Writing—original draft; investigation; methodology; formal analysis; validation; writing—review and editing. **Jinliang Liang**: data curation; writing—review and editing; investigation. **Yihang Gong**: Data curation; writing—review and editing; investigation. **Cuicui Xiao**: Data curation; Investigation; Writing—review and editing. **Jianye Cai**: Writing—review and editing; project administration. **Tingting Wang**: Writing—review and editing; project administration. **Qiang You**: Project administration; writing—review and editing. **Jiebin Zhang**: Writing—review and editing; project administration. **Haitian Chen**: Writing—review and editing; visualization; investigation. **Jiaqi Xiao**: Writing—review and editing; visualization; investigation. **Yingcai Zhang**: Writing—review and editing; project administration. **Yang Yang**: Project administration; supervision; writing—review and editing. **Hua Li**: Conceptualization; funding acquisition; writing—review and editing; project administration; resources; supervision. **Jia Yao**: Conceptualization; funding acquisition; writing—review and editing; project administration; resources; supervision. **Qi Zhang**: Conceptualization; funding acquisition; writing—review and editing; project administration; resources; supervision. **Jun Zheng**: Conceptualization; supervision; funding acquisition; project administration; writing—review and editing; resources.

## CONFLICT OF INTEREST STATEMENT

The authors declare no conflict of interest.

## ETHICS STATEMENT

This study was approved by the Institutional Animal Care and Use Committee (IACUC), Jennio Biotech Co., Ltd. (Approval No. JENNIO‐IACUC‐2024‐A041).

## Supporting information


**Figure S1:** Median coefficient of variation (CV) of protein quantification across organs.
**Figure S2:** Proteomic features in relation to multi‐organ aging.
**Figure S3:** Tissue‐shared differentially expressed proteins (DEPs) across tissues.
**Figure S4:** Significant plasma‐tissue pairwise correlations.
**Figure S5:** Longitudinal expression dynamics of complement proteins in healthy individuals over an actionable time frame.
**Figure S6:** Unique and shared organ‐resolved proteins.
**Figure S7:** Impact of aging on the metabolite composition and inter‐organ correlations in diverse tissue types.
**Figure S8:** Microbial diversity and taxonomic profiling of gut microbiota in young and aged mice.
**Figure S9:** Taxonomical difference, contig assembly, and functional pathway genes of aging‐related gut microbiota.
**Figure S10:** Comparison of the relative level of MEs for PM1‐PM12 between the young and aged groups.
**Figure S11:** Comparison of the relative level of MEs for PM13‐PM24 between the young and aged groups.
**Figure S12:** Comparison of the relative level of MEs for MM1‐MM14 between the young and aged groups.
**Figure S13:** Protein‐metabolite interaction network based on WGCNA modules.

**Table S1:** Significant plasma‐tissue pairwise correlated proteins.
**Table S2:** Organ‐resolved and aging‐related proteins across tissues.

## Data Availability

The data that support the findings of this study are available from the National Genomics Data Center. Restrictions apply to the availability of these data, which were used under license for this study. Data are available from the author(s) with the permission of the National Genomics Data Center. The data reported in this paper have been deposited in the Genome Sequence Archive (metagenomics, CRA023889) and OMIX (metabolomics, OMIX009232) in the National Genomics Data Center, China National Center for Bioinformation/Beijing Institute of Genomics, Chinese Academy of Sciences under BioProject accession number PRJCA032056 (https://ngdc.cncb.ac.cn/bioproject/browse/PRJCA032056). The mass spectrometry proteomic data have been deposited to the ProteomeXchange Consortium (https://proteomecentral.proteomexchange.org) via the iProx partner repository with the data set identifier PXD061948. The data and scripts used are saved in GitHub (https://github.com/JenverZhang/CrossTissueAging). Supplementary materials (figures, methods, tables, graphical abstract, slides, videos, Chinese translated version, and update materials) may be found in the online DOI or iMeta Science http://www.imeta.science/.
