# Supplementary material for: Integrative cross‐tissue analysis unveils complement‐immunoglobulin augmentation and dysbiosis‐related fatty acid metabolic remodeling during mammalian aging
Source: Imeta. 2025 Apr 12;4(3):e70027. doi: 10.1002/imt2.70027 (PMC12130579; doi:10.1002/imt2.70027)
Supplement: Supplementary file 1 — Figure S1: Median coefficient of variation (CV) of protein quantification across organs. Figure S2: Proteomic features in relation to multi‐organ aging. Figure S3: Tissue‐shared differentially expressed proteins (DEPs) across tissues. Figure S4: Significant plasma‐tissue pairwise correlations. Figure S5: Longitudinal expression dynamics of complement proteins in healthy individuals over an actionable time frame. Figure S6: Unique and shared organ‐resolved proteins. Figure S7: Impact of aging on the metabolite composition and inter‐organ correlations in diverse tissue types. Figure S8: Microbial diversity and taxonomic profiling of gut microbiota in young and aged mice. Figure S9: Taxonomical difference, contig assembly, and functional pathway genes of aging‐related gut microbiota. Figure S10: Comparison of the relative level of MEs for PM1‐PM12 between the young and aged groups. Figure S11: Comparison of the relative level of MEs for PM13‐PM24 between the young and aged groups. Figure S12: Comparison of the relative level of MEs for MM1‐MM14 between the young and aged groups. Figure S13: Protein‐metabolite interaction network based on WGCNA modules. [file IMT2-4-e70027-s001.docx]

**Supporting Information to**

**Integrative cross-tissue analysis unveils complement-immunoglobulin augmentation and dysbiosis-related fatty acid metabolic remodeling during mammalian aging**

Feng Zhang^1,2,3#^, Rong Li^2#^, Yasong Liu^1,2#^, Jinliang Liang^2#^, Yihang Gong^1,2,3^, Cuicui Xiao^4^, Jianye Cai^5^, Tingting Wang^1,2^, Qiang You^1,2^, Jiebin Zhang^1,2^, Haitian Chen^1,2^, Jiaqi Xiao^1,2^, Yingcai Zhang^1,2,6^, Yang Yang^1,2^, Hua Li^1,2*^, Jia Yao^1,2*^, Qi Zhang^3*^, Jun Zheng^1,2*^

^1^Department of Hepatic Surgery and Liver Transplantation Center of the Third Affiliated Hospital of Sun Yat-sen University; Organ Transplantation Research Center of Guangdong Province, Guangdong Province Engineering Laboratory for Transplantation Medicine. Guangzhou 510630, China

^2^Guangdong Provincial Key Laboratory of Liver Disease Research, the Third Affiliated Hospital of Sun Yat-sen University, Guangzhou 510630, China

^3^Biotherapy Center & Cell-gene Therapy Translational Medicine Research Center, the Third Affiliated Hospital of Sun Yat-sen University, Guangzhou 510630, China

^4^Department of Anesthesiology, the Third Affiliated Hospital of Sun Yat-sen University, Guangzhou 510630, China

^5^Comprehensive transplant center, Feinberg school of medicine, Northwestern University, Chicago, 60611, USA

^6^Department of Hepatobiliary Surgery, People's Hospital of Xinjiang Uyghur Autonomous Region, Urumqi 830001, China

^#^These authors contributed equally: Feng Zhang, Rong Li, Yasong Liu, and Jinliang Liang

*Correspondence authors: lihua3@mail.sysu.edu.cn (Hua Li); [yaojia6@mail.sysu.edu.cn](mailto:yaojia6@mail.sysu.edu.cn) (Jia Yao); [zhangq27@mail.sysu.edu.cn](mailto:zhangq27@mail.sysu.edu.cn) (Qi Zhang); [zhengj67@mail2.sysu.edu.cn](mailto:zhengj67@mail2.sysu.edu.cn) (Jun Zheng)


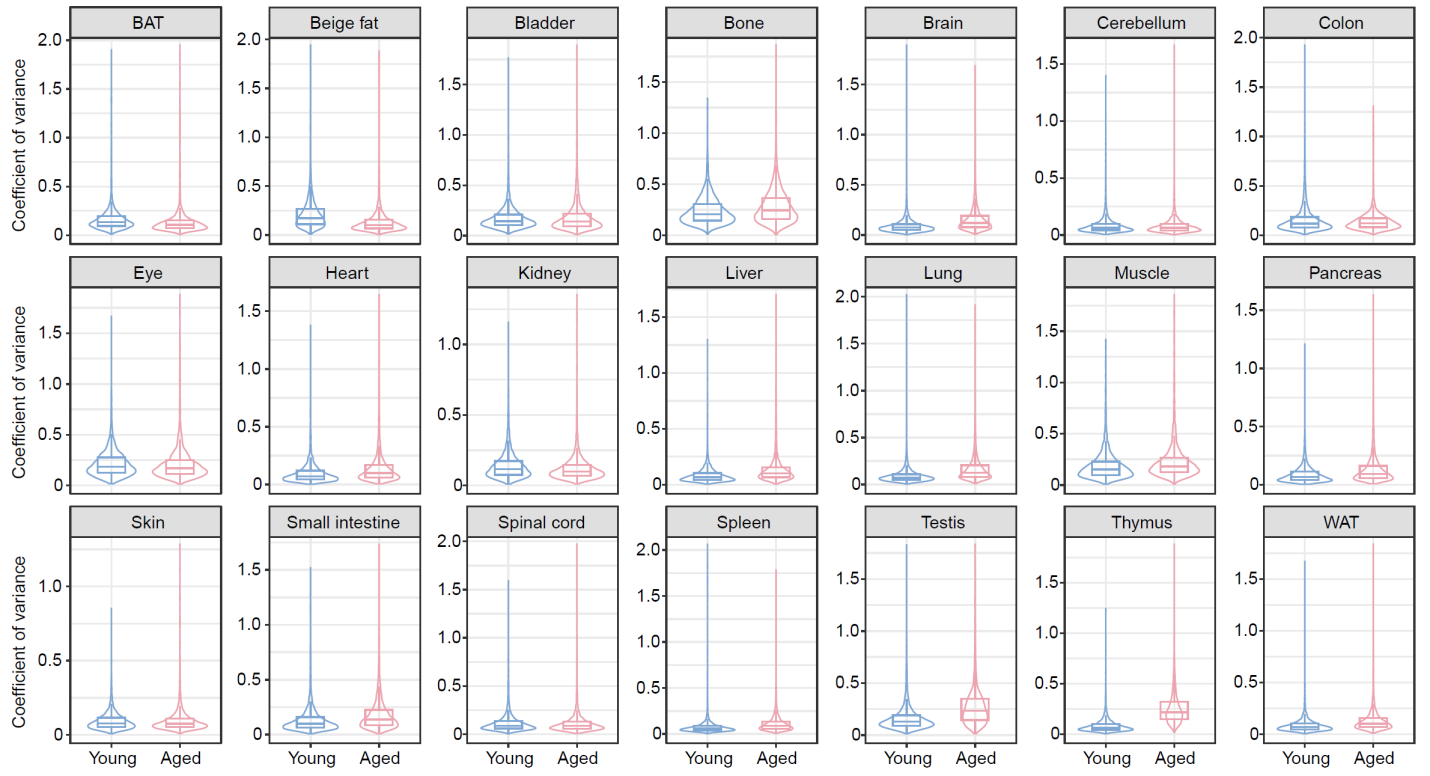


**Figure S1 Median coefficient of variation (CV) of protein quantification across organs.** The comparable median CV across biological replicates in each organ, calculated by the normalized quantified proteins in the proteomic profiles.


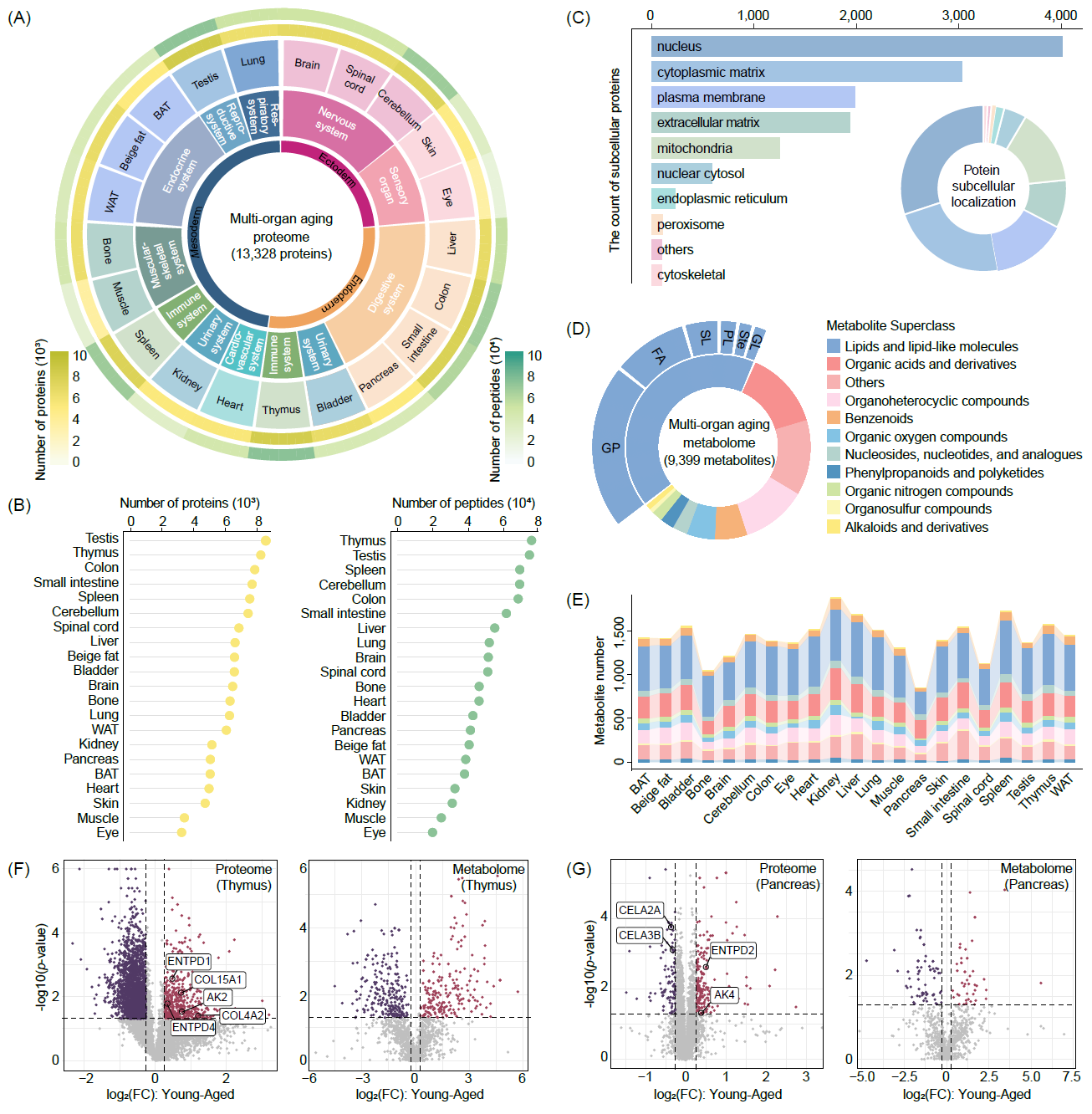


**Figure S2 Proteomic features in relation to multi-organ aging.** (A-B) The total number of proteins and peptides detected in young and aged tissue covering various biological systems. *n* = 4 mice per group. (C) The total count and proportion of subcellular location of proteins detected in the proteomics. (D-E) The proportion (D) and number (E) of various metabolite superclass detected in separate tissue from young and aged mice. *n* = 4 mice per group. FA, fatty acids; GL, glycerolipids; GP, glycerophospholipids; SL, saccharolipids; Ste, sterol lipids; PL, phospholipids. (F-G) Volcano plots showing differentially expressed proteins (left) or metabolites (right) in young versus aged thymus (F) or pancreas (G), with selected features labeled. Dark purple indicates that a feature was upregulated in young mice relative to aged mice, deep red indicates upregulation in aged mice relative to young mice and grey indicates no significant change in regulation. FC, fold change.


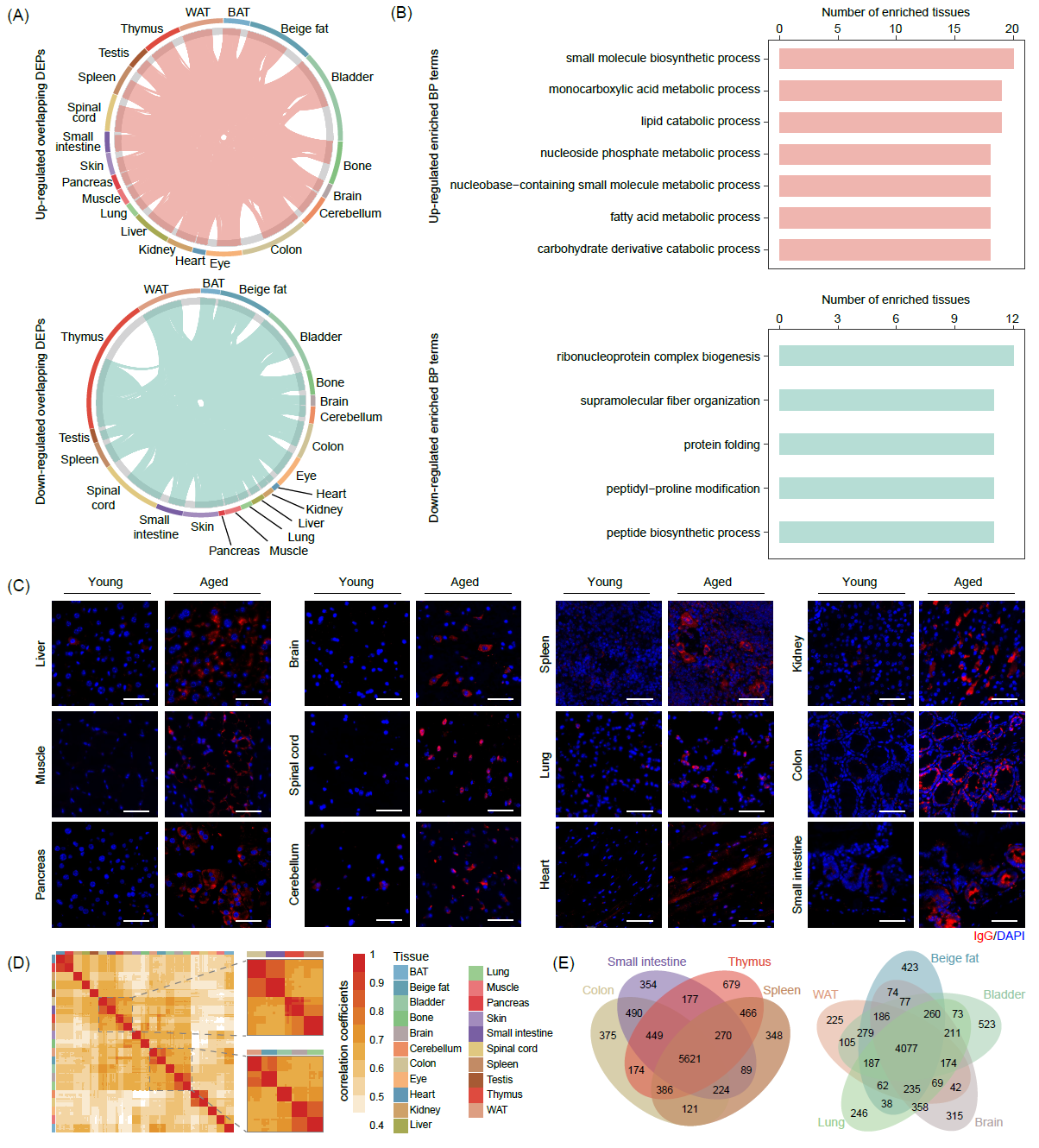


**Figure S3 Tissue-shared differentially expressed proteins (DEPs) across tissues.** (A) The immense inter-tissue overlaps in aging-accumulated (top) and aging-reduced (bottom) DEPs among multiple tissues. (B) GO enrichment analyses of tissue-overlapping aging-accumulated (top) and aging-reduced (bottom) DEPs. (C) Immunofluorescence staining of IgG expression in the young (2-month-old) and aged (20-month-old) male C57BL/6 mice across 12 tissues (*n* = 4 mice per group). Scale bars, 50 μm. (D) Heatmap displaying the mutual Spearman’s correlation within each organ, with two major organ communities clustering with similar proteomic expressions profiles (Spearman, rho > 0.6, *p* < 0.05), named as Cluster 1 (composed of the colon, small intestine, thymus, and spleen) and Cluster 2 (composed of WAT, beige fat, bladder, brain, and lung). (E) Venn diagram of co-expressed protein intersection within four organs of Cluster 1 (left) and five organs of Cluster 2 (right), separately.


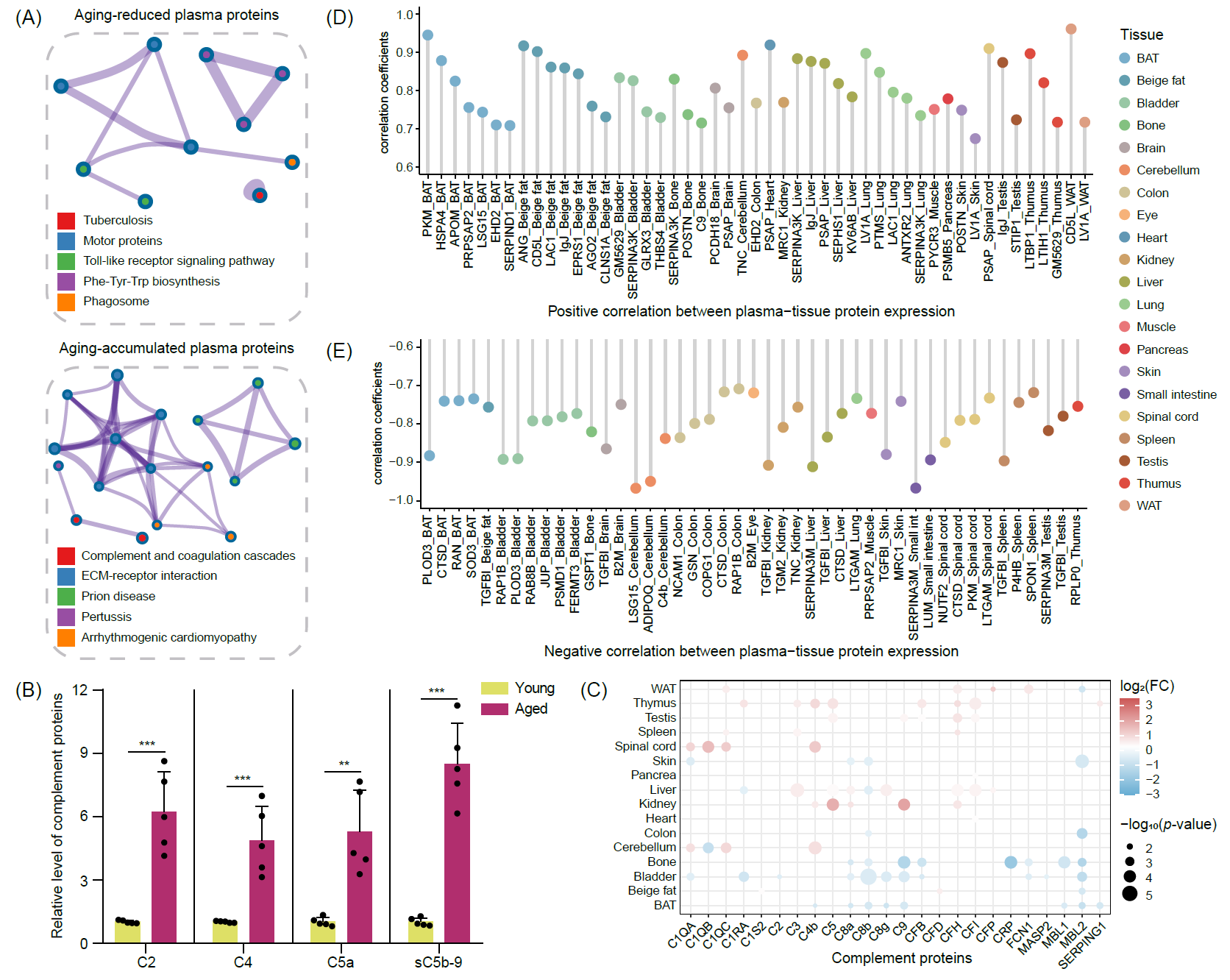


**Figure S4 Significant plasma-tissue pairwise correlations.** (A) Pathway enrichment network of aging-reduced (top) and aging-accumulated (bottom) plasma proteins. Each term is represented by a circular node sized according to the number of input proteins it contains, with color indicating cluster identity. Terms with a similarity score > 0.3 are connected by edges, where edge thickness reflects the similarity score. (B) Enzyme-Linked Immunosorbent Assay (ELISA) test to detect the C2, C4, C5a and sC5b-9 levels in the plasma from young and aged mice (*n* = 5 mice per group). (C) Bubble plot showing the change of multiple complement proteins expression across tissues between young and aged mice. (D-E) Lollipop charts demonstrating specific proteins with significantly positive (D) or negative (E) plasma-tissue pairwise correlations.


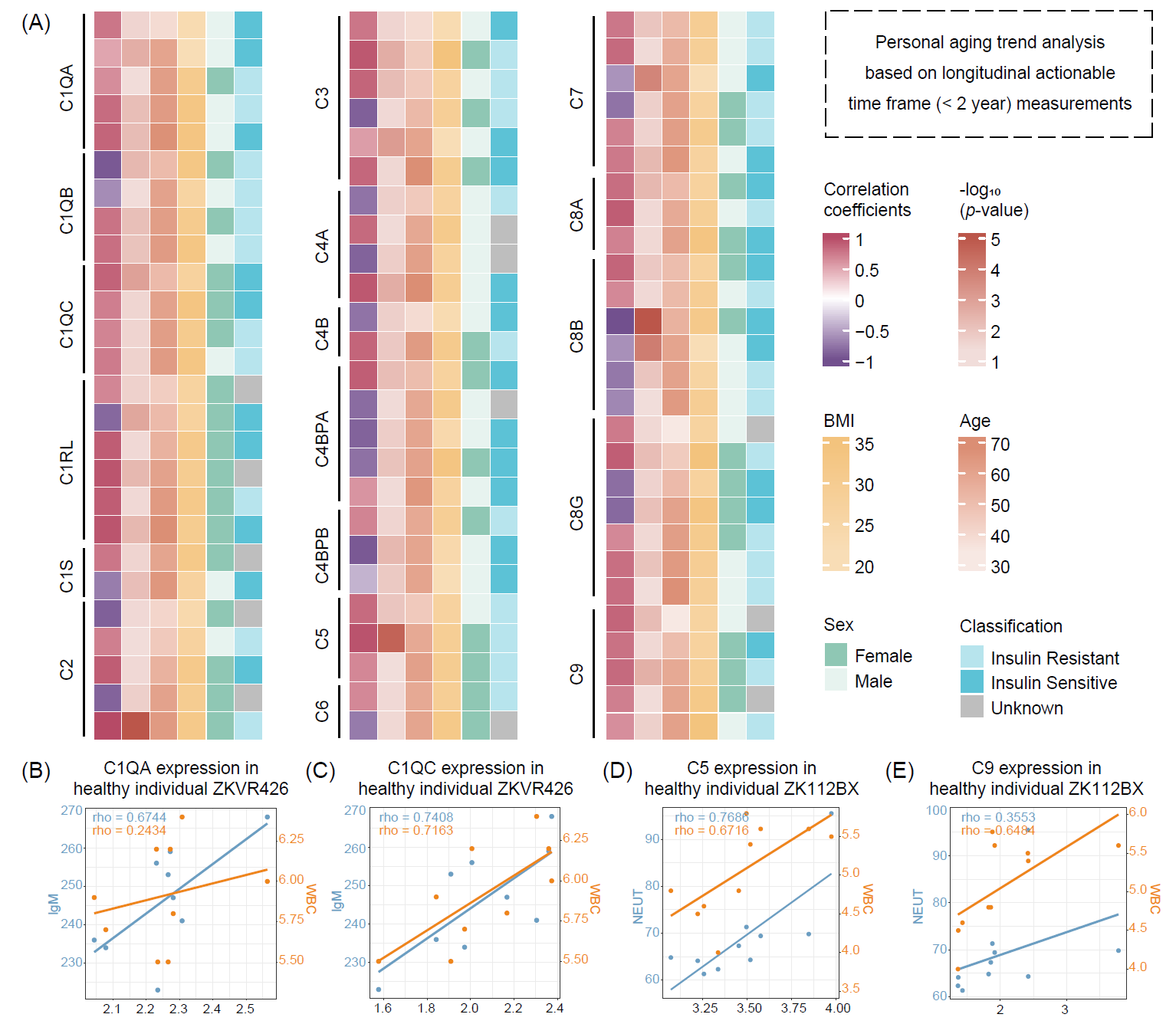


**Figure S5 Longitudinal expression dynamics of complement proteins in healthy individuals over an actionable time frame.** (A) Heatmap showing Spearman correlation between complement protein expression and actionable-time aging in individuals with at least five healthy visits spanning 700 days to 2 years. (B-C) Longitudinal expression profiles of C1QA (B) and C1QC (C) in healthy individual ZKVR426 over nine visits within a 2-year period were associated with levels of IgM and WBC amounts. (D-E) Longitudinal expression profiles of C5 (D) and C9 (E) in healthy individual ZK112BX over ten visits within a 2-year period were associated with levels of NEUT and WBC amounts. NEUT, neutrophils; WBC, white blood cell.


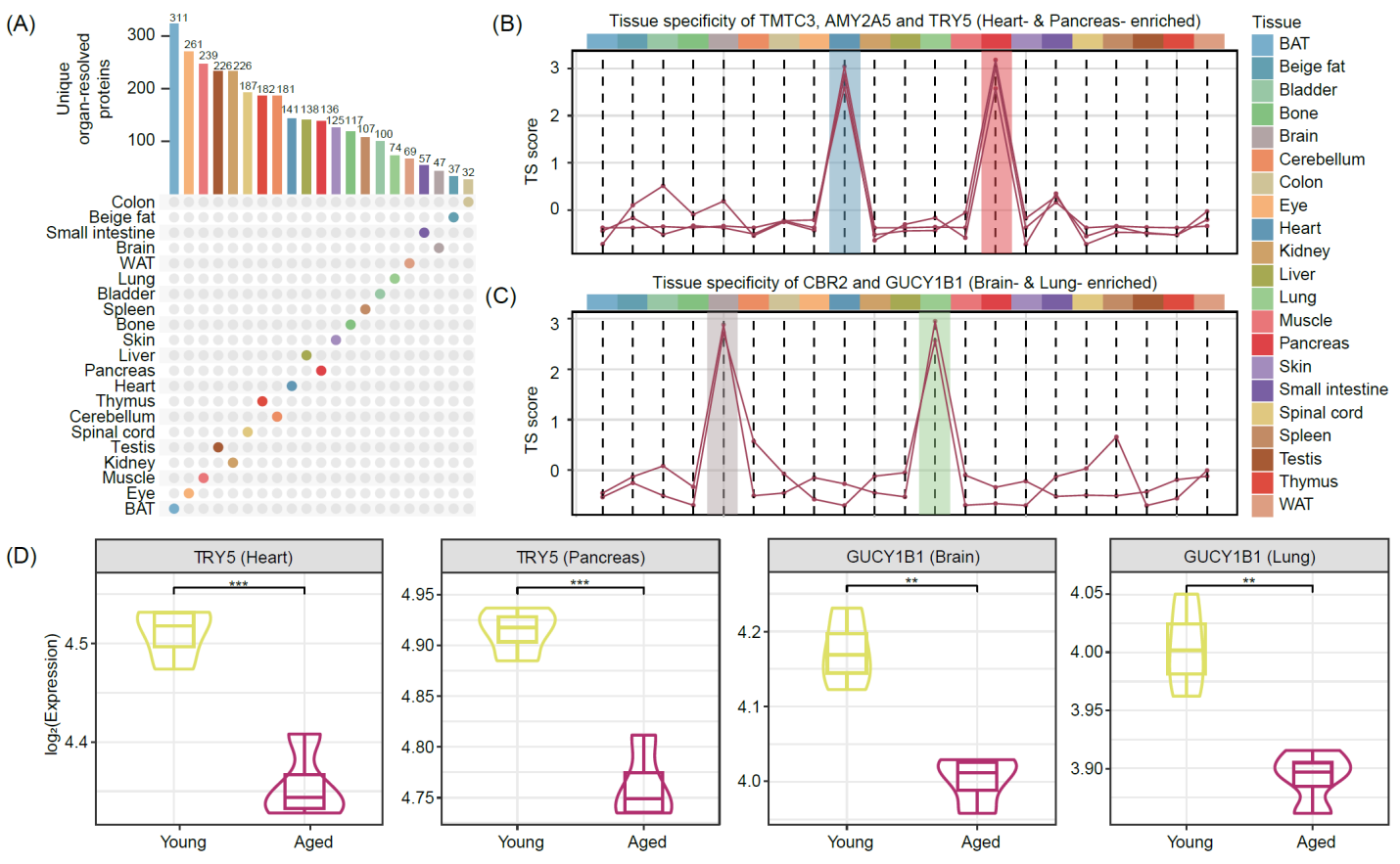


**Figure S6 Unique and shared organ-resolved proteins.** (A) The count of unique organ-resolved proteins in each organ. (B) TS score distributions indicate heart- & pancreas-enrichment of TMTC3, AMY2A5 and TRY5. (C) TS score distributions indicate brain- & lung-enrichment of CBR2 and GUCY1B1. (D) Significant age differences of TRY5 varying in heart & pancreas, as well as GUCY1B1 varying in brain & lung.


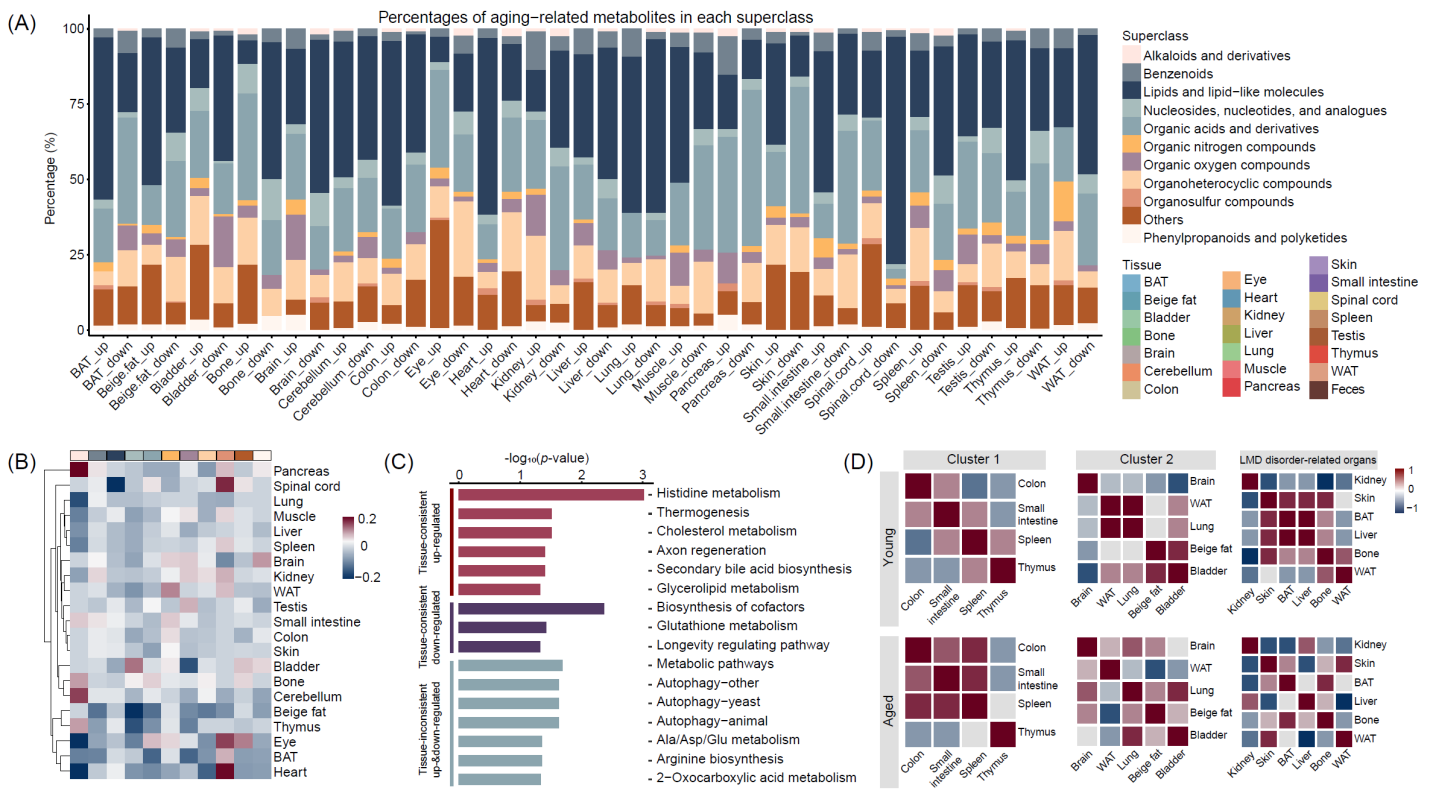


**Figure S7** **Impact of aging on the metabolite composition and inter-organ correlations in diverse tissue types.** (A) Tissue-resolved and age-resolved proportions of metabolite superclass. (B) Unsupervised clustering heatmap showing aging-related change score of different metabolite classes in each tissue. (C) KEGG pathway enrichment analysis of tissue-consistent upregulated metabolites, tissue-consistent downregulated metabolites and tissue-inconsistent dysregulated metabolites. (D) The modification of inter-organ correlation among Cluster 1, Cluster 2 and LMD disorders-related organs in the aged versus young group. LMD disorders, lipid metabolism dysfunction-related disorders.


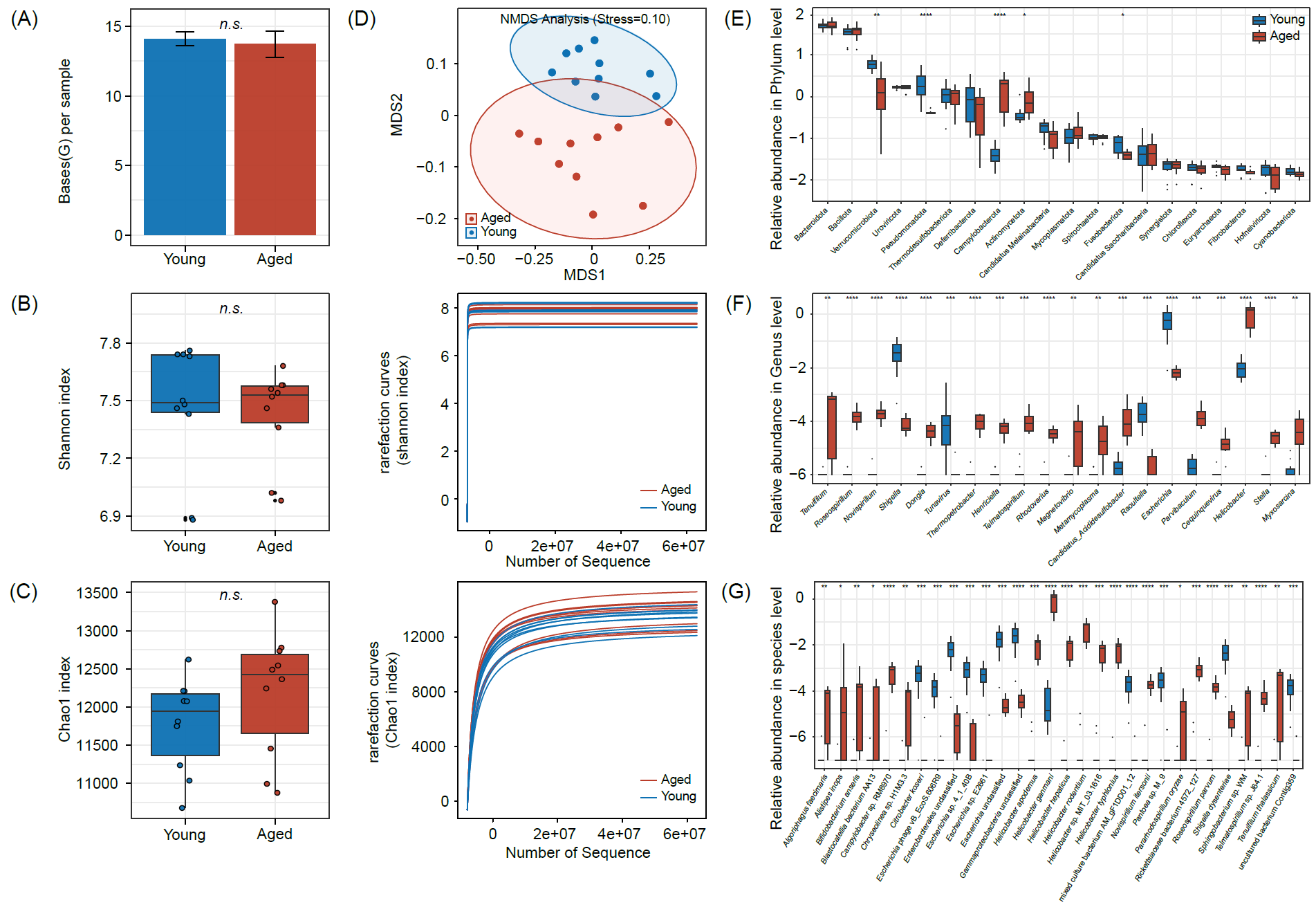
**Figure S8 Microbial diversity and taxonomic profiling of gut microbiota in young and aged mice.** (A) Comparison of the shotgun metagenomic sequencing depth of microbial communities in fecal samples from the young and aged mice (*n* = 10 mice per group). Data was presented as mean ± standard deviation (SD). (B-C) Comparison of the Shannon index (B, left), Chao1 index (C, left), and corresponding refraction curves (B and C, right) of the gut microbiota in the young and aged group. (D) Non-metric multidimensional scaling (NMDS) analysis based on the species relative abundances in the young and aged groups. (E-G) Comparison of the relative abundances of the top 20 phyla (E), top 20 genera (F) and top 30 species (G) with the most significant differences between the young and aged groups.


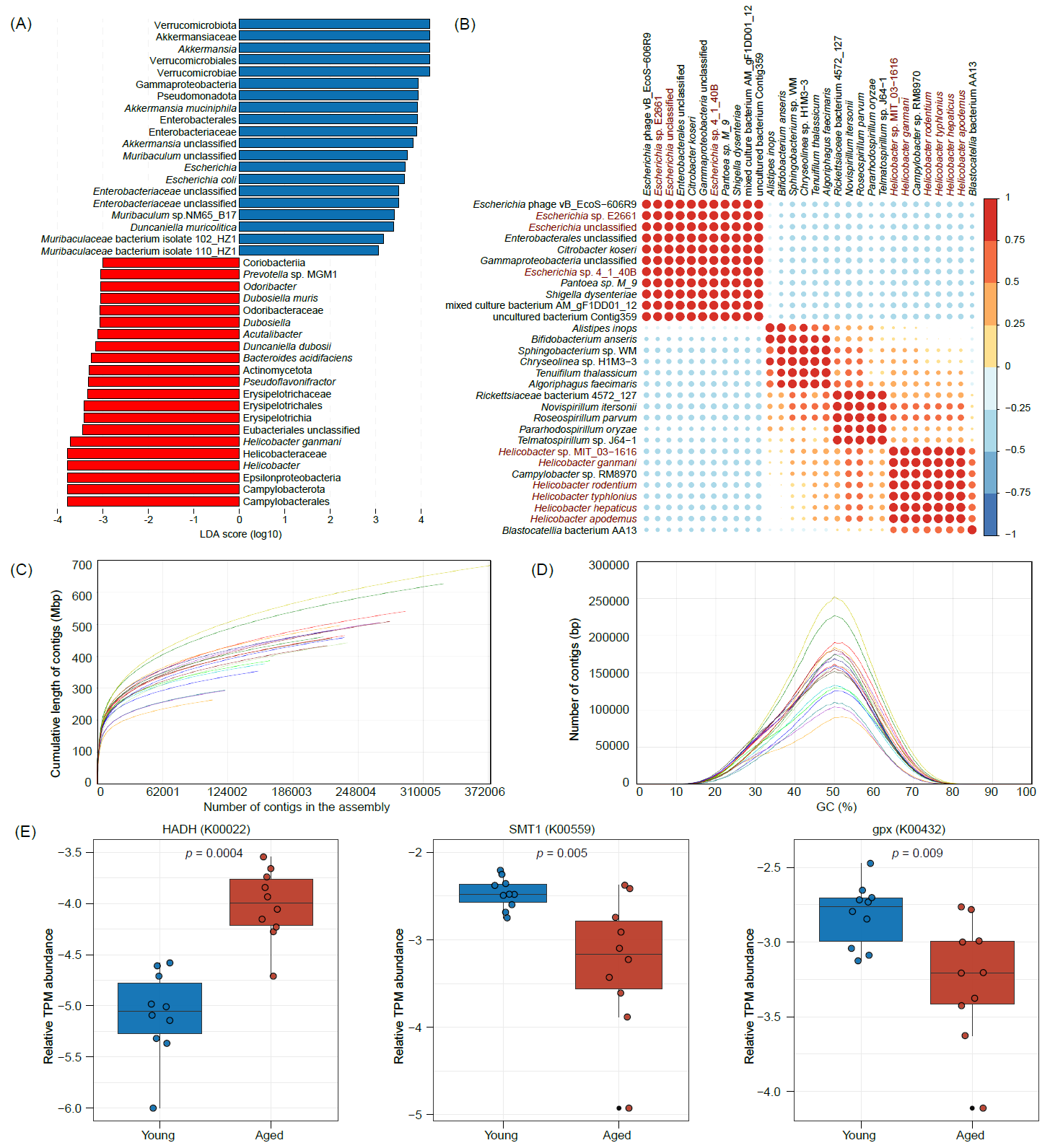


**Figure S9 Taxonomical difference, contig assembly, and functional pathway genes of aging-related gut microbiota.** (A) Significant age-related taxonomic difference identified by Linear discriminant analysis effect size (LEfSe) algorithm with Linear Discriminant Analysis (LDA) score > 3 or < -3. (B) Clustering correlation heatmap of the top 30 differentially abundant gut microbe’s species. (C) The contig assembly of shotgun metagenomic sequencing data from microbial communities in fecal samples of both young and aged mice was performed using Quast. (D) Waveform chart displaying the number of contigs with Guanine-Cytosine (GC) percentages within a specific range. (E) Comparison of the levels of representative genes associated with lipid metabolic dysfunction in feces of young and aged groups.


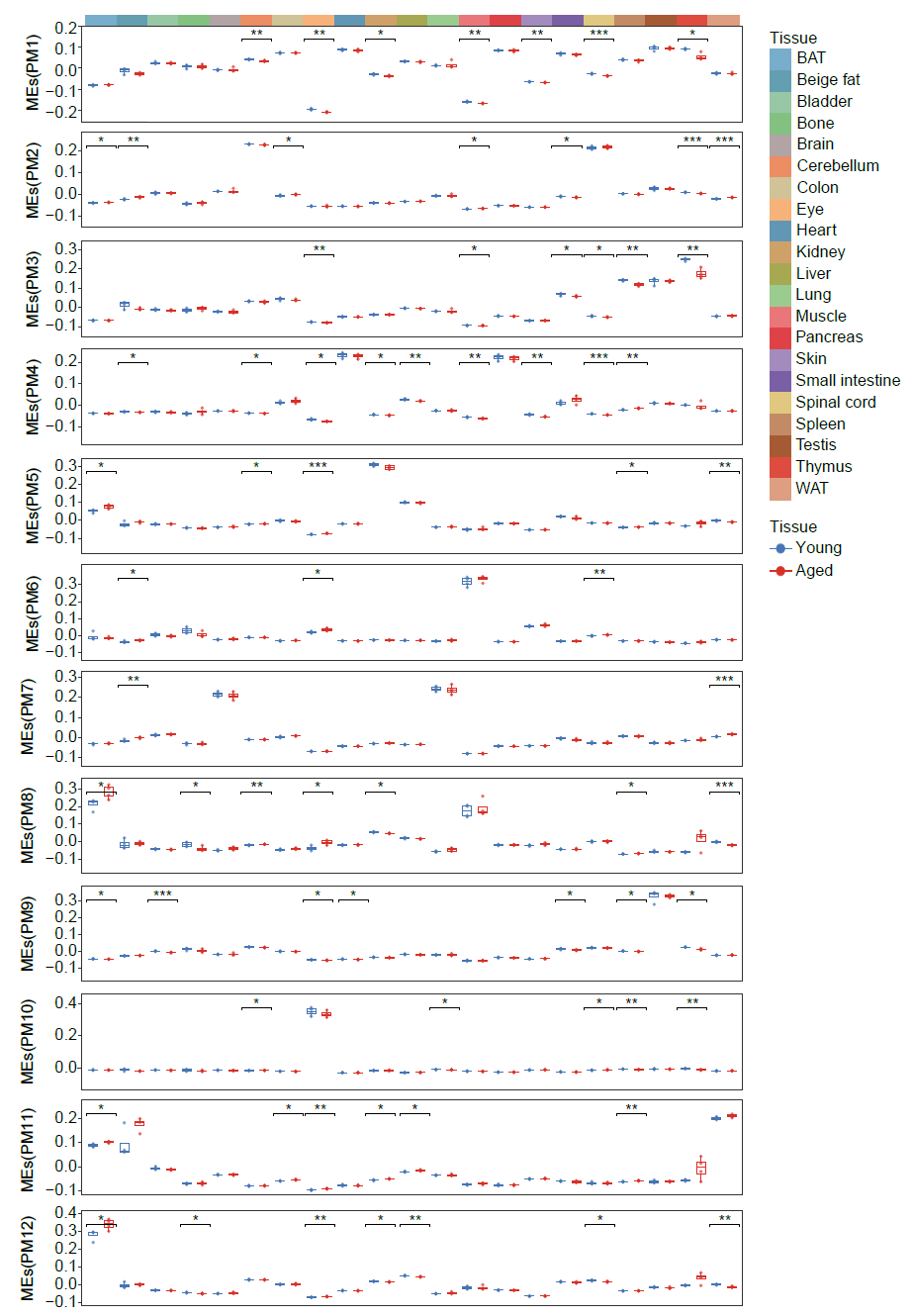


**Figure S10 Comparison of the relative level of MEs for PM1-PM12 between the young and aged groups.**


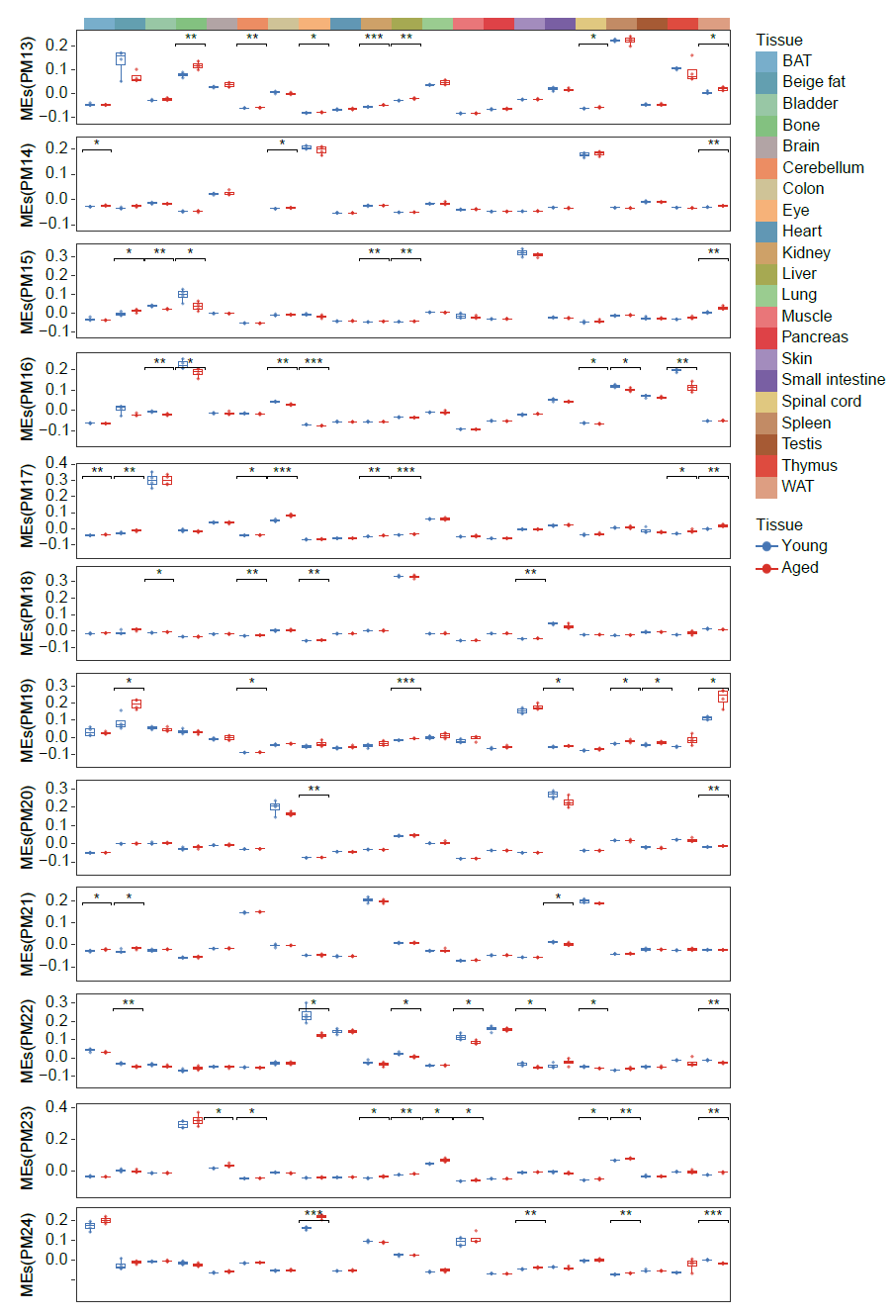


**Figure S11 Comparison of the relative level of MEs for PM13-PM24 between the young and aged groups.**
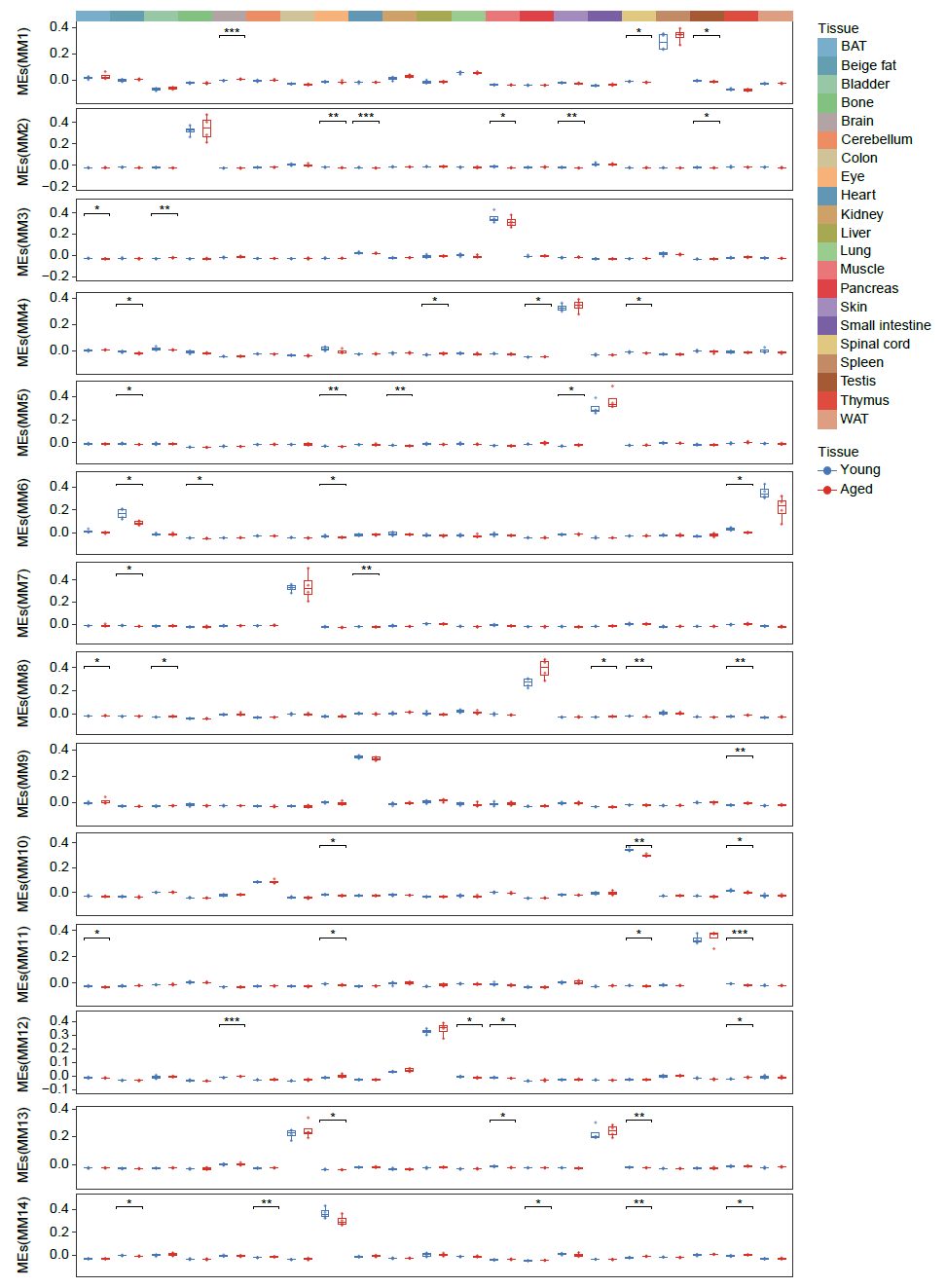


**Figure S12 Comparison of the relative level of MEs for MM1-MM14 between the young and aged groups.**


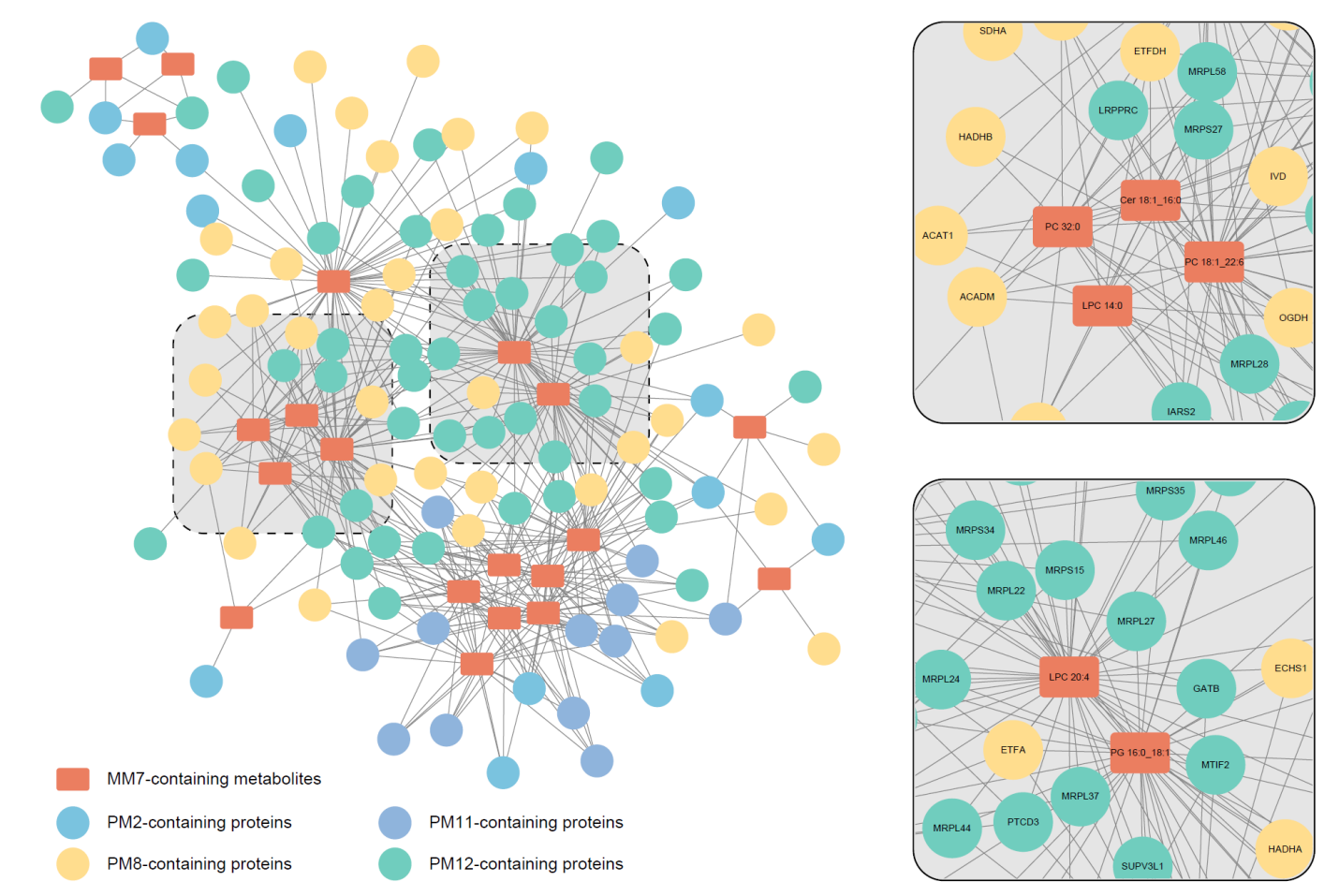


**Figure S13 Protein-metabolite interaction network based on WGCNA modules.** The network was established based on PM2/8/11/12 and MM7 (left). Significant correlations between omega-3 fatty acids (PC 18:1_22:6) and HADHB, omega-6 fatty acids derivative (LPC 20:4) and HADHA were observed (right).
